# Two oppositely-charged *sf3b1* mutations cause defective development, impaired immune response, and aberrant selection of intronic branch sites in *Drosophila*

**DOI:** 10.1371/journal.pgen.1009861

**Published:** 2021-11-01

**Authors:** Bei Zhang, Zhan Ding, Liang Li, Ling-Kun Xie, Yu-Jie Fan, Yong-Zhen Xu

**Affiliations:** 1 University of Chinese Academy of Sciences, Beijing, China; 2 Key Laboratory of Insect Developmental and Evolutionary Biology, Center for Excellence in Molecular Plant Sciences, Chinese Academy of Sciences; Shanghai, China; 3 RNA Institute, State Key Laboratory of Virology, Hubei Key Laboratory of Cell Homeostasis, College of Life Science, Wuhan University, Hubei, China; Albert Einstein College of Medicine, UNITED STATES

## Abstract

SF3B1 mutations occur in many cancers, and the highly conserved His662 residue is one of the hotspot mutation sites. To address effects on splicing and development, we constructed strains carrying point mutations at the corresponding residue His698 in *Drosophila* using the CRISPR-Cas9 technique. Two mutations, *H698D* and *H698R*, were selected due to their frequent presence in patients and notable opposite charges. Both the *sf3b1-H698D* and–*H698R* mutant flies exhibit developmental defects, including less egg-laying, decreased hatching rates, delayed morphogenesis and shorter lifespans. Interestingly, the *H698D* mutant has decreased resistance to fungal infection, while the *H698R* mutant shows impaired climbing ability. Consistent with these phenotypes, further analysis of RNA-seq data finds altered expression of immune response genes and changed alternative splicing of muscle and neural-related genes in the two mutants, respectively. Expression of *Mef2-RB*, an isoform of *Mef2* gene that was downregulated due to splicing changes caused by *H698R*, partly rescues the climbing defects of the *sf3b1-H698R* mutant. Lariat sequencing reveals that the two *sf3b1-H698* mutations cause aberrant selection of multiple intronic branch sites, with the *H698R* mutant using far upstream branch sites in the changed alternative splicing events. This study provides in vivo evidence from *Drosophila* that elucidates how these SF3B1 hotspot mutations alter splicing and their consequences in development and in the immune system.

## Author summary

In the past decade, one of the important findings in the RNA splicing field has been that somatic SF3B1 mutations widely occur in many cancers. Including R625, H662, K666, K700 and E902, there are five hotspot mutation sites in the highly conserved HEAT repeats of SF3B1. Several kinds of H662 mutations have been found widely in MDS, AML, CLL and breast cancers; however, it remains unclear how these H662 mutations alter splicing and whether they have in vivo effects on development. To address these questions, in this manuscript, we first summarized the H662 mutations in human diseases and constructed two corresponding *Drosophila* mutant strains, *sf3b1-H698D* and *-H698R* using CRISPR-Cas9. Analyses of these two fly strains find that the two oppositely charged *Sf3b1-H698* mutants are defective in development. In addition, one mutant has decreased climbing ability, whereas the other mutant has impaired immune response. Further RNA-seq allows us to find responsible genes in each mutant strain, and lariat sequencing reveals that both mutations cause aberrant selection of the intronic branch sites. Our findings provide the first in vivo evidence that *Sf3b1* mutations result in defective development, and also reveal a molecular mechanism of these hotspot histidine mutations that enhance the use of cryptic branch sites to alter splicing. Importantly, we demonstrate that the *H698R* mutant prefers to use far upstream branch sites.

## Introduction

Pre-mRNA splicing, catalyzed by the spliceosome, a large and dynamic complex consisting of five snRNAs and >100 proteins, is critical for eukaryotic gene expression and regulation [Reviewed in 1,2]. Human disease mutations in *trans*-acting splicing factor genes and *cis*-acting pre-mRNA sequences can alter or disrupt splicing and drive the development of cancers [Reviewed in 3,4–8]. Mutations in nearly twenty splicing factors connected with cancers, are highly recurrent in myeloid malignancies [9–14], chronic lymphocytic leukemia (CLL) [15–18] and uveal melanoma (UVM) [19,20], and also frequently occur in bladder carcinoma [21], breast cancers [22,23], lung adenocarcinoma [21,24] and pancreatic ductal adenocarcinoma [25].

Over the past decade, SF3B1, SRSF2, U2AF1 and ZRSR2, splicing factors that are involved in early intron selection and pre-spliceosome assembly, have been identified as the most frequently mutated splicing factors in cancers based on the fast-developing next-generation sequencing techniques [9,10,13,16,26]. Somatic mutations in SF3B1 are particularly prevalent in myelodysplastic syndromes (MDS) [9–11,26] and CLL [16,18], as well as in other solid tumors, such as UVM, pancreatic ductal adenocarcinomas and breast cancers [19,20,27].

SF3b is a 450-kDa hetero-heptameric protein complex and a major component of 17S U2 snRNP [28]. Studies in yeast and human have revealed that the SF3b complex is required for the formation of pre-spliceosomal complex A [29,30] and directly interacts with the intronic branch site (BS) and flanking RNA sequences [31,32]. After the U4/U6-U5 tri-snRNP joins in, the SF3b complex is released from the complex B^act^ [33]. As the largest subunit of SF3b, SF3B1 has 20 highly conserved HEAT repeats in its C-terminus, and U2AF1/2- and SF3b14a-interacting motifs in its N-terminus [29,34–37]. SF3B1 also interacts with the splicing factors Prp5 [28,38,39], SUGP1 [40] and Prp3 [41].

Most of the SF3B1 mutations are located in its HEAT repeats, especially in HEATs 4–12, where five residues, R625, H662, K666, K700 and E902, are hotspots and most of them exhibit cancer lineage specificities [21,42,43]. Mutation K700E is linked with blood cancers [44], K666N is linked with AML [45], R625 mutations are linked with UVM [20,27,46], and E902 mutations are linked with bladder urothelial carcinoma (BLCA) [21]. However, H662 mutations have no obvious correlation with cancer-types, being found in MDS, AML, CLL, UVM and breast cancers [9,10,47–52]. Recent cryo-EM structures have identified intermolecular hydrogen bonds between the intronic polypyrimidine tract and most of the hotspot residues in several spliceosomal complexes [53–55], and those mutations are also in the proximity of Prp5’s highly-conserved DPLD motif in the human 17S U2 snRNP [28]. In addition, the SF3B1 mutations in blood cancers destabilize the SF3B1—SUGP1 interaction in humans [40], and their equivalent mutations in the yeast SF3B1-homolog Hsh155 lead to altered Hsh155—Prp5 interaction [38,56].

Transcriptome analyses of human cell lines and mouse models have found that alternative 3′ splice site (A3SS) events are enriched in SF3B1 mutation-mediated splicing changes, in which upstream cryptic 3′SSs are preferentially used [40,47,57–59]. Several mechanistic models have been proposed for this alteration: i) mutated SF3B1 facilitates selection of cryptic 3′SSs by either overcoming certain steric hindrance within a region downstream of BS [57], enhancing interactions of SF3B1 with specific nucleotides flanking the upstream BS [47] or increasing recognition of inaccessible 3′SSs buried in RNA secondary structures [58]; ii) mutated SF3B1 induces a conformational change in the U2 snRNP complex leading to the selection of a stronger upstream BS [59]; and iii) mutated SF3B1 disrupts its interaction with SUGP1 and facilitates BS recognition to use a cryptic 3′SS [40].

*Sf3b1* mutations have also been studied for genetic interactions and for the sensitization of clinically relevant drugs, as well as the developmental and splicing defects in *C*. *elegans* [60]. In zebrafish, studies of *Sf3b1* mutants have revealed that Sf3b1 is essential for the neural crest development [61] and the hematopoietic differentiation [62], and regulates erythroid maturation and proliferation via TGFβ signaling [63]. However, *sf3b1* mutants in *Drosophila* have not been previously investigated.

Several H662 mutations, including H662D and H662R, two oppositely-charged residues, have been found in multiple cancers without obvious type preference as mentioned above. However, it remains unclear how these H662 mutations alter splicing and whether they have in vivo effects on development. To test these mutations in an in vivo model system, we constructed two mutant fly strains, *sf3b1*-*H698D* and -*H698R*, in which the homologous residue His698 in *Drosophila* Sf3b1 was mutated to Aspartic acid (D) and Arginine (R), respectively. Both mutants exhibit defects in development; the *H698D* mutant has an impaired immune response to fungal infection, whereas the *H698R* mutant has a decreased ability in climbing. RNA-seq allowed us to find responsible candidate genes in each mutant, and lariat sequencing revealed that both mutations cause aberrant selection of branch sites and that the *H698R* mutant prefers to use upstream branch sites.

## Results

### Construction of *sf3b1-H698* mutant flies by CRISPR-Cas9 system

**Fig 1:**
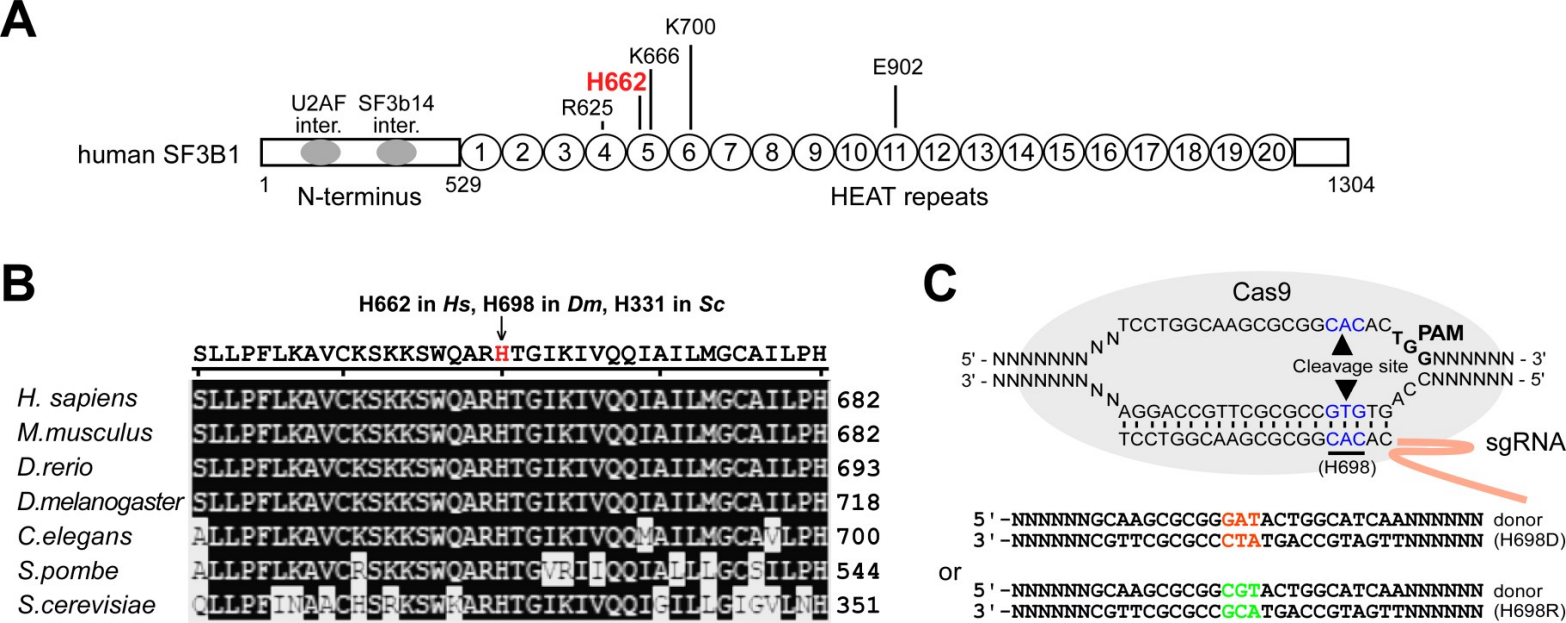
Construction of *sf3b1-H698D* and *-H698R* mutant strains. (**A**) Frequent mutations of SF3B1 in human cancers. The most frequently mutated five sites, motifs and domains of human SF3B1 are indicated. (**B**) Alignment of SF3B1 homologous proteins from the yeast to human. The conserved histidine residue, amino acid position 662 in humans and 698 in *Drosophila*, is indicated. (**C**) Schematics for the construction of *sf3b1-H698D* and *-H698R* mutant strains using a CRISPR/cas9 system. Mutated sequences for coding are in orange and green, and the WT histidine sequence is in blue.

H662 mutations had been widely identified in patients as one of the five hotspots in SF3B1 (Fig 1A). There may be additional mutational hotspots, but they have not yet been experimentally validated for their contribution to disease or splicing changes. We collected 34 available online reports in cancers and found five residue mutations of H662, including Q, D, R, Y, and N (S1 Table and refs therein). This histidine residue is invariant in all species (Fig 1B). To address the mechanism of altered splicing by mutations of this highly conserved residue and their effects on development, we constructed *Drosophila* strains with mutations at H698, the corresponding residue of human H662, using the CRISPR-Cas9 system. Since our previous data in yeast demonstrated that the D and R mutations of Hsh155/SF3B1 result in opposite splicing effects on branch site mutant reporters and opposite effects on interaction with Prp5 [38], two mutant strains, *sf3b1-H698D* and *-H698R*, were constructed (Fig 1C). The *sf3b1*-H698 homozygous mutants were successfully obtained and confirmed by genomic PCRs and Sanger sequencing (S1A Fig). In comparison to the *WT* strain, the mRNA and protein levels of *sf3b1* and their cellular localizations were not detectably changed in the two mutant strains (Figs S1B and 1C).

### *sf3b1-H698* mutants are defective in development

**Fig 2:**
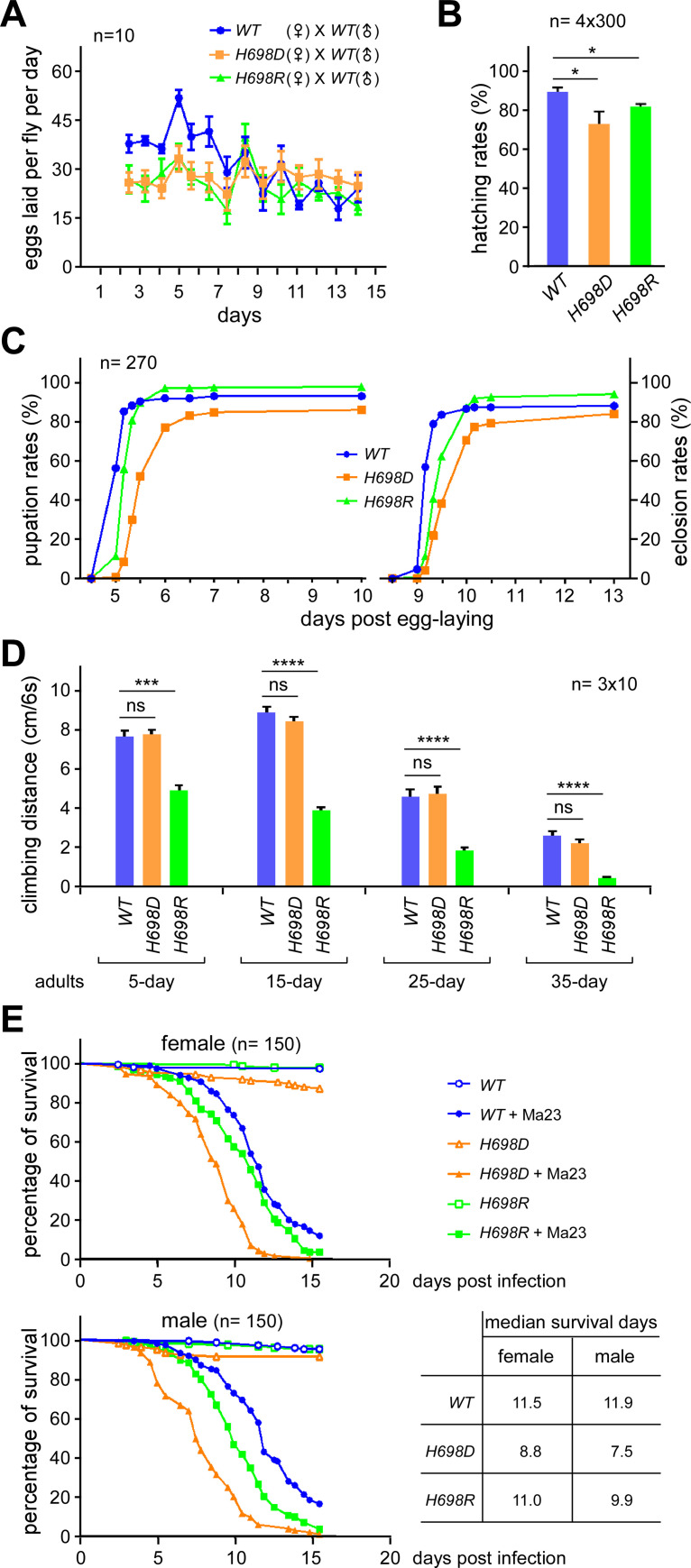
*sf3b1* mutant strains are defective in reproduction, development and fungi-infection resistance. (**A**) Fewer eggs were laid in the early stage by *sf3b1-H689D* and -*H698R* mutants. Fecundity was measured over a period of 12 days from females crossed with the *WT* males, and each time point represents data from ten female adults. Data of females crossed with males from their own strain are shown in S2A Fig. Total eggs laid (per fly) of females crossed with the *WT* males are shown in S2B Fig. (**B**) Decreased hatching rates of *H698D* and *H698R* mutants. Statistical differences were determined according to the t-test. The hatching rates of *WT*, *sf3b1-*H698D and -H698R are 89.6%, 73.3%, 82.1%, respectively. The graph was shown by mean ± SEM, and *p* values were 0.0452 for *H698D* and 0.0211 for *H698R*. (**C**) Developmental stages were elongated for *sf3b1* mutant strains. Time point started from the first day after egg-laying. (**D**) Decreased climbing ability of the *sf3b1-H698R* strain, but not the -*H698D* strain. Adults at four ages were assessed. Statistical data are shown as mean ± SEM, *: *p* < 0.05, **: *p* < 0.01, ***: *p* < 0.001, ns: no significance. *H698D*-5d vs *WT*-5d: p > 0.9999, *H698R*-5d vs *WT*-5d: p < 0.0001, *H698D*-15d vs *WT*-15d: p = 0.3401, *H698R*-15d vs *WT*-15d: p < 0.0001, *H698D*-25d vs *WT*-25d: p = 0.9323, *H698R*-25d vs *WT*-25d: p < 0.0001, *H698D*-35d vs *WT*-35d: p = 0.3259, *H698R*-35d vs *WT*-35d: p < 0.0001. (**E**) Survival time courses of *Drosophila* adults post infection with *M*. *anisopliae*. The median survival days was measured and is listed on the right. Female: *WT*-LT50 vs *H698D*-LT50: P = 0.000, *WT*-LT50 vs *H698R*-LT50: P = 0.006; Male: *WT*-LT50 vs *H698D*-LT50: P = 0.000, *WT*-LT50 vs *H698R*-LT50: P = 0.008.

To investigate the effects of *sf3b1*-H698 mutations on development, a variety of phenotypes were tested, including fecundity, hatching, pupation, eclosion and lifespan. In comparison to the *WT*, females of the two *sf3b1* mutants laid significantly fewer eggs, roughly 35% fewer in the first 5-days (Figs 2A, S2A and S2B). Embryonic development of the two mutants was also impaired, showing 10–20% decreased hatching rates (Fig 2B). The development of both mutants was obviously retarded in metamorphosis; both mutants exhibited less pupation and eclosion rates during the first 36 hours, *H698D* being worse than *H698R* (Fig 2C). Furthermore, the lifespan of the two mutants was significantly shortened, going from the *WT*’s median of 72 days to a median of 58 days (S2C Fig). Taken together, these phenotypes demonstrate that the H698 homozygous mutations in Sf3b1 result in defective development of *Drosophila*, with *H698D* being slightly more defective than *H698R*. We had preliminarily tested their heterozygous mutants, neither of which exhibited a notable phenotype; therefore, homozygous mutants were used for further investigations in this study.

### *H698R* is defective in climbing, *H698D* is defective in immune response

When culturing fly strains, we noticed that the movement of the *sf3b1-H698R* mutant was obviously different from the other strains. Therefore, a climbing assay was performed for the adult flies [64]. Compared to the *WT*, the *H698R* adults exhibited a significantly decreased ability in climbing, which worsened when they were older. In contrast, the climbing ability of the *H698D* adults was not significantly changed for all four tested ages (Fig 2D).

We also investigated fungal infection of the mutants. After infection with *Metarhizium anisopliae* ARSEF 23 (*Ma23*), survival was significantly impaired for *H698D* females and males, but not significantly changed for *H698R* females and only slightly decreased for *H698R* males (Fig 2E), suggesting that resistance to fungal infection is reduced in the *H698D* mutant.

These results reveal that the two kinds of H698 mutations have different impacts on *Drosophila* movement and immune systems, suggesting that downstream genes of *sf3b1*, such as muscle, neuron or immunity-related genes, are affected differently by H698D and H698R mutations in Sf3b1.

### Innate immune response genes are affected in the *sf3b1* mutants

**Fig 3:**
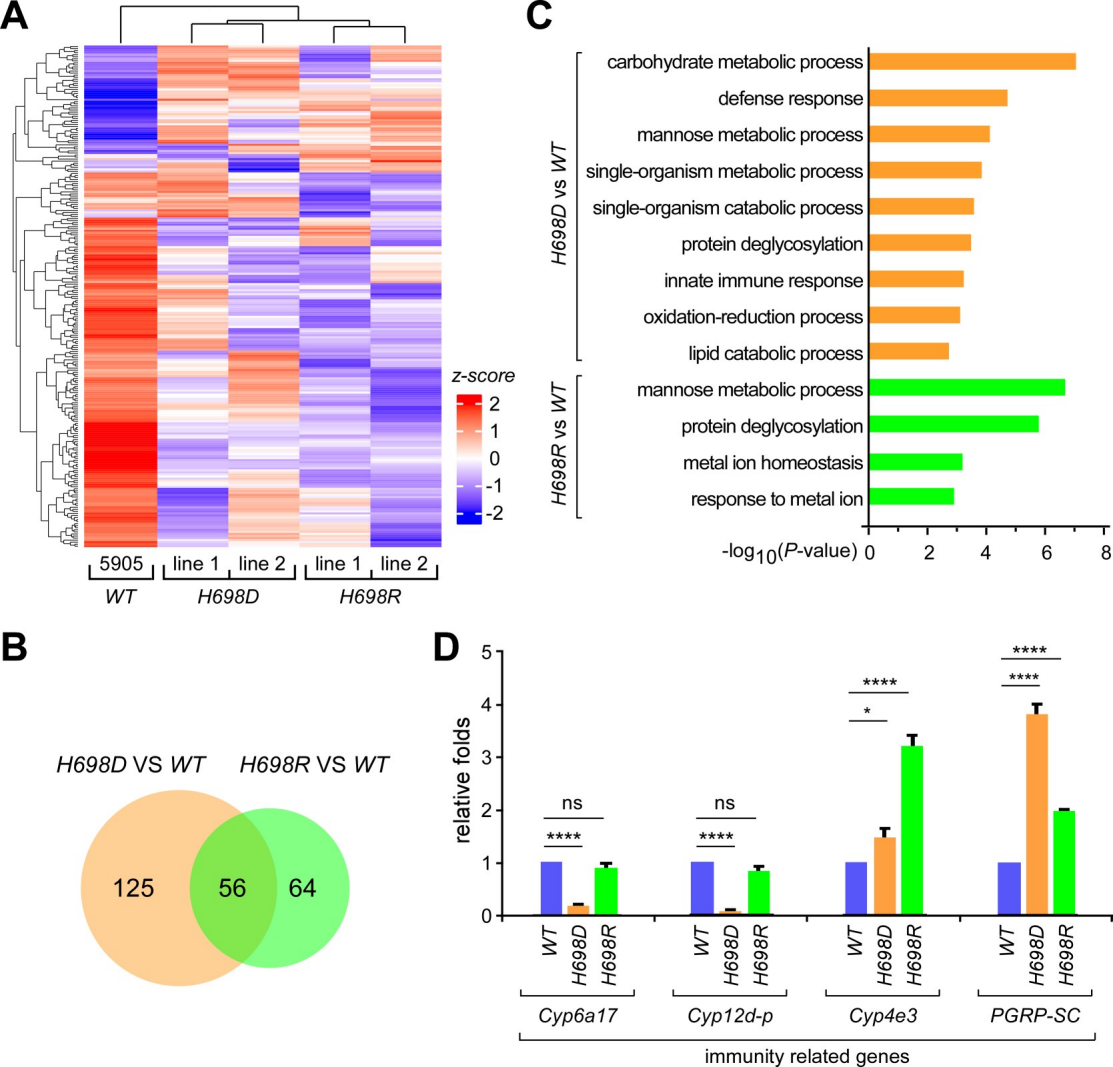
Differentially expressed genes in the two *sf3b1* mutants. (**A**) Heat map of the differentially expressed genes. Compared to the *WT* strain, the expression of 181 genes in *H698D* and 120 in *H698R* were significantly changed (*p* < 0.05), those are analyzed and presented in the map. (**B**) Overlap of differentially expressed genes in the two mutant strains. (**C**) GO enrichment of differentially expressed genes. Enrichment in *H698D* and *H698R* is shown in two distinct groups. (**D**) Validation of differentially expressed genes by qRT-PCR. Statistical data are shown as mean ± SEM, n = 3, two-tailed unpaired Student’s t-test.

We then performed mRNA-seq of the *WT* and mutant fly adults (5d), in which two lines of each *sf3b1* mutant were sequenced for accuracy (S2 Table). Correlation analyses of the two lines from each mutant strain suggested that they are highly consistent with each other (S3 Fig). Expression levels of 181 genes were significantly changed in both lines of the *H698D* mutant, and 120 genes were significantly changed in both lines of the *H698R* mutant; and 56 of them were shared in the *H698D* and *H698R* (Fig 3A and 3B and S3 Table). Further GO analyses indicated that genes in the mannose metabolic process and protein deglycosylation were highly enriched in the two mutants, whereas the innate immune response genes were enriched only in *H698D* (Fig 3C). This is consistent with the above findings that both mutants are defective in development and that the *H698D* mutant is sensitive to fungal infection (Fig 2).

To validate this, we performed RT-qPCRs to test levels of the innate immune response genes, including *Cyp6a-17*, *Cyp12d-p*, *Cyp4e3* and *PGRP-SC*. The mRNA levels of *Cyp4e3* and *PGRP-SC* were increased in both mutants, while those of *Cyp6a-17* and *Cyp12d-p* were dramatically decreased in *H698D* but not in *H698R* (Fig 3D). These data are consistent with the bioinformatic analyses, and suggest that the decreased fungal resistance of *H698D* would be due to the affected expression of cytochrome P450 (CYP) family genes that are involved in the detoxification of foreign compounds, such as the tested *Cyp6a-17* and *Cyp12d*, both of which are known to have monooxygenase activity and are induced by xenobiotic treatment [65].

### Differential AS events in the two mutants

**Fig 4:**
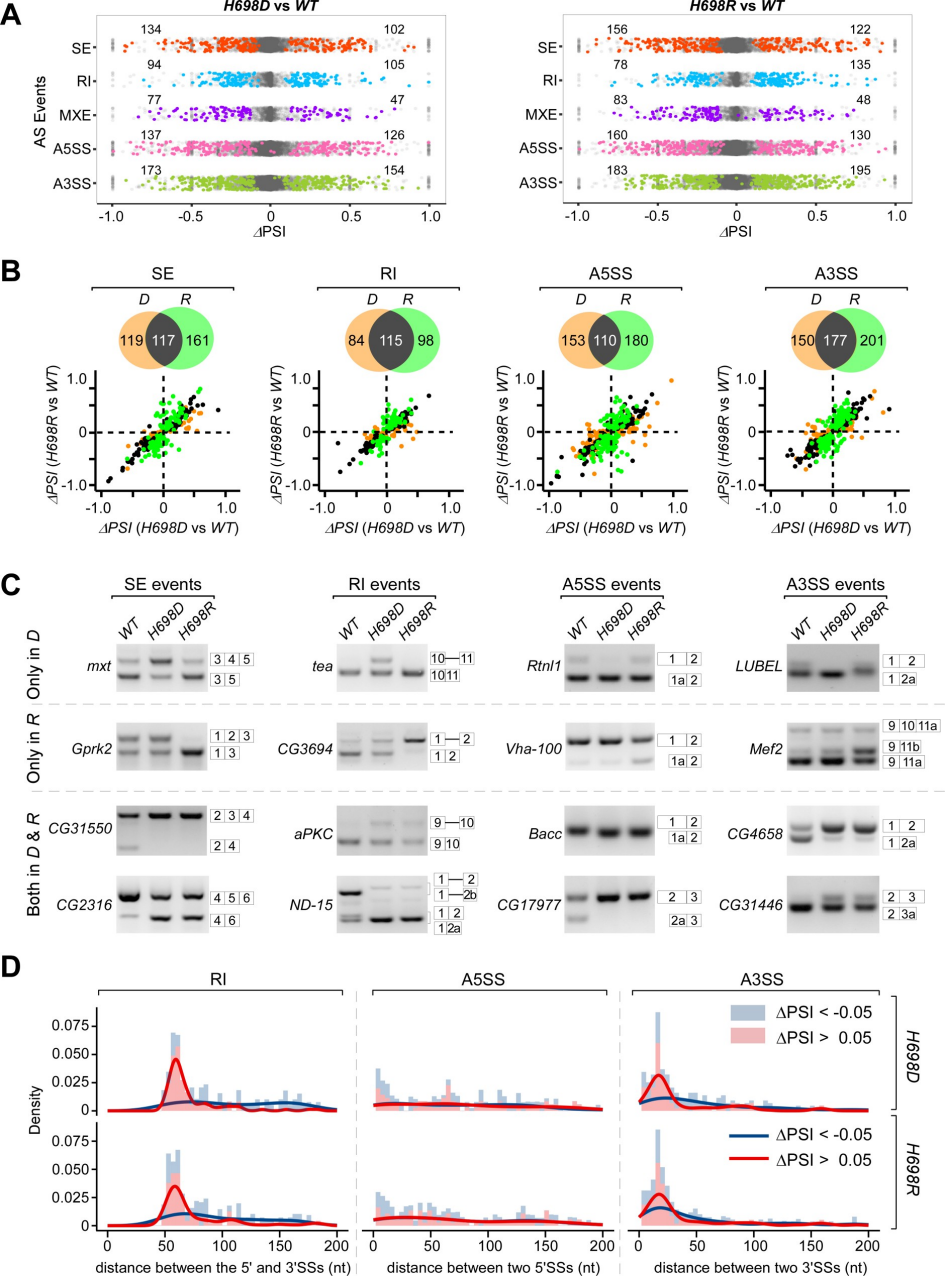
Alternative splicing changes in the *sf3b1* mutant strains. (**A**) Scatter plots of ΔPSI of all splicing events between the two mutants and the *WT*, respectively. Using rMATS, AS events were analyzed in five types. Significantly changed events (|ΔPSI| > 0.05, FDR < 0.05, and with supporting reads > = 5) are shown in color dots. (**B**) Scatter plots of significantly changed AS events between the two mutants. Four types of AS were analyzed. Black dots: overlapped events in both mutants, brown dots: unique in *H698D*, green dots: unique in *H698R*. Significantly changed AS: |ΔPSI| > 0.05, FDR < 0.05. (**C**) Validation of AS changes in the two *sf3b1* mutant strains by RT-PCR. Overlapped and unique events were selected from the four analyzed AS types. AS isoforms are indicated on the right side of gels. (**D**) Distribution of distance between two splice sites of the significant changed AS events in *sf3b1* mutants. For the RI events, those two are the 5′ and 3′SSs from the intron; for the A5SS events, those are the two alternative 5′SSs; for the A3SS, those are the two alternative 3′SSs.

Using the common differential AS (DS) analysis tool rMATS, we identified 1,149 and 1,290 AS events that were significantly changed in the two mutants, respectively (Figs 4A and S4, and S4 Table). The most common belong to two groups, alternative 3′SS (A3SS) and 5′SS (A5SS); the other three groups (SE, skipped exon; RI, retained intron; MXE, mutually exclusive exons) were relatively less common. In each group, ~40–60% DS events were shared by the two mutants (Fig 4B). To validate this, we performed RT-PCRs of randomly picked events that are either shared or unique; all of them were consistent with our bioinformatic analysis (Figs 4C and S5).

Furthermore, we analyzed the distribution of distances between two SSs of the DS events in the RI, A5SS and A3SS groups. First, distance between two SSs in the RI group, or the length of the intron, was highly enriched in a segment of 50–80 nt whose splicing was enhanced by both *sf3b1* mutants (ΔPSI > 0.05), whereas no such enrichment was seen in inhibited RI events (ΔPSI < -0.05) (Fig 4D left). Second, in the A3SS group, the distance between two alternative 3′SSs was enriched in a segment of 6–30 nt in both *sf3b1* mutants (Fig 4D right). In contrast, the distance between the two alternative 5′SSs in the A5SS group was not obviously enriched (Fig 4D middle). These results suggest that the two *sf3b1-H698* mutants not only share similar effects on alternative splicing events but also have different unknown specificities in the recognition of pre-mRNA substrates.

### *H698R*-specifically changes AS events in muscle and neural-related genes

**Fig 5:**
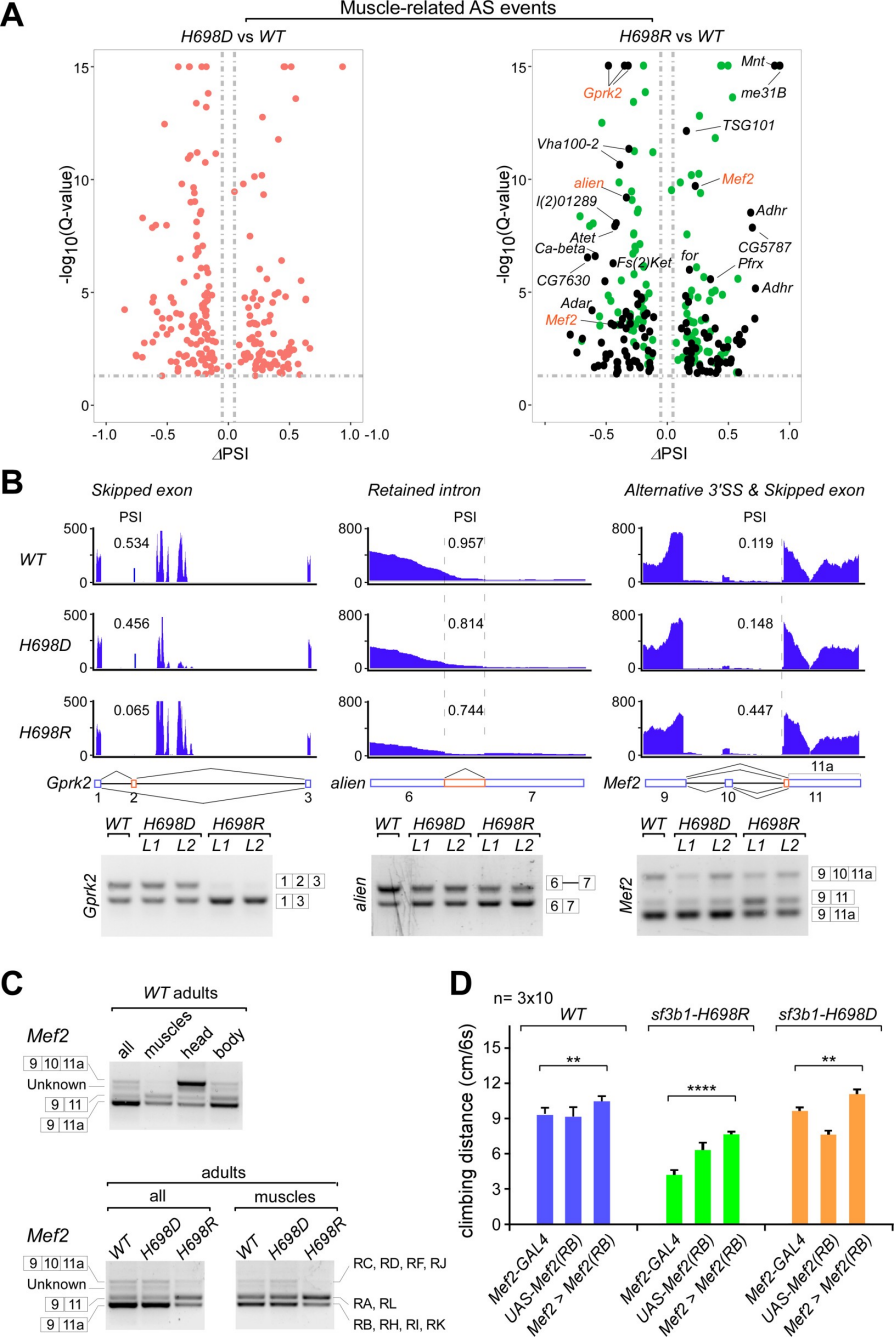
Alternative splicing changes involved in the muscle development in the *H698R* mutant. (**A**) Scatter plots of significant DS events involved in the muscle development in *sf3b1* mutants. *H698R*-specifically changed AS events in the muscle-related genes were analyzed. DS events in *H698D* (red), *H698R* (green), and unique in *H698R* (black) are indicated in dots. (**B**) Validation of *H698R*-unique DS events by RT-PCR. PSI values for DS events (red rectangles) in each fly strain are indicated. (**C**) Tissue-specific AS events in *Mef2* were changed in the *H698R* mutant. Whole adults, muscle (flight muscle + jump muscle), head and body (thorax + abdomen) from *Drosophila* strains were analyzed. Ligation of exons and their corresponding isoforms are indicated, details of the 11 alternative isoforms information are listed in S8 Fig. (D) Expression of the isoform RB of *Mef2* partly rescued the climbing defects of *sf3b1-H698R*. The *Mef2-GAL4* was used to drive expression of transgenic *UAS-Mef2(RB)*. RB is one of the multiple AS isoforms of *Mef2* (S8 Fig), and downregulated in the *sf3b1-H698R* mutant. Statistical data are shown as mean ± SEM, **: *p* < 0.01, ****: *p* < 0.0001. For H698R, *Mef2-GAL4 vs Mef2 > Mef2(RB)*: p < 0.0001. For H698D, *GAL4 vs Mef2 > Mef2(RB)*: p = 0.0052.

To find affected candidate genes that may cause the climbing defects of the *H698R* flies (Fig 2D), we compared DS events between the two mutants for genes that are functionally involved in muscle and neuron development [66,67]. In comparison to *H698D*, 125 DS events from 70 muscle-related genes and 70 DS events from 42 neural-related genes are specific to the *H698R* mutant (S5 Table). Of these, AS of *Gprk2* [68,69], *alien* [70] and *Mef2* [71–73], three genes that are important for *Drosophila* locomotion [67], were significantly changed in *H698R*, but not in *H698D* (Figs 5A, S6, S7A and S7C); these were further validated by RT-PCR analyses (Fig 5B). Interestingly, the identified DS events of the *Mef2* gene are tissue specific, showing different AS patterns in the muscle (including the flight, jump and part of leg muscles), head and body from the *WT* adult (Fig 5C upper); the AS products with ligation of exons 9+11a in muscles were dramatically decreased in *H698R* but not in *H698D* (Fig 5C lower). These data suggest that the reduced climbing ability could be caused by changed AS events from a number of muscle and neural genes, which would be due to cryptic splicing elements that can be recognized by *Sf3b1*-*H698R* but not by *Sf3b1*-*H698D*, or alternatively could be due to the altered genes having regular elements that are recognized by *Sf3b1*-WT and *Sf3b1*-*H698D*, but cannot be recognized by *Sf3b1*-*H698R*.

### Expression of the *Mef2-RB* isoform partly rescues the climbing defects of *sf3b1-H698R*

Alternative splicing of *Mef2* is complicated in *Drosophila*, generating 11 isoforms according to our RNA-seq data and Flybase annotations (S7C and S8 Figs). To address whether it is the changed AS of *Mef2* that contributes to the climbing defects, according to annotations of tissue-specific expression in Flybase, we determined that the isoform RB is the most likely downregulated transcript in the *sf3b1-H698R* mutant flies. Therefore, we constructed transgenic flies of *Mef2* promoter-driven *GAL4* and *UAS*-driven *Mef2(RB)* in the *WT* and *sf3b1-H698* mutant backgrounds, respectively. Additional expression of the *Mef2(RB)* isoform doubled the climbing ability of the *H698R* mutant, while only ~15% increase of the climbing ability of the *WT* and *H698D* mutant (Fig 5D), suggesting that the impaired locomotion activity of the *H698R* mutant is at least partly due to the changed AS of the *Mef2* gene.

### Changed recognition of 5′SSs and 3′SSs results in different types of DS

**Fig 6:**
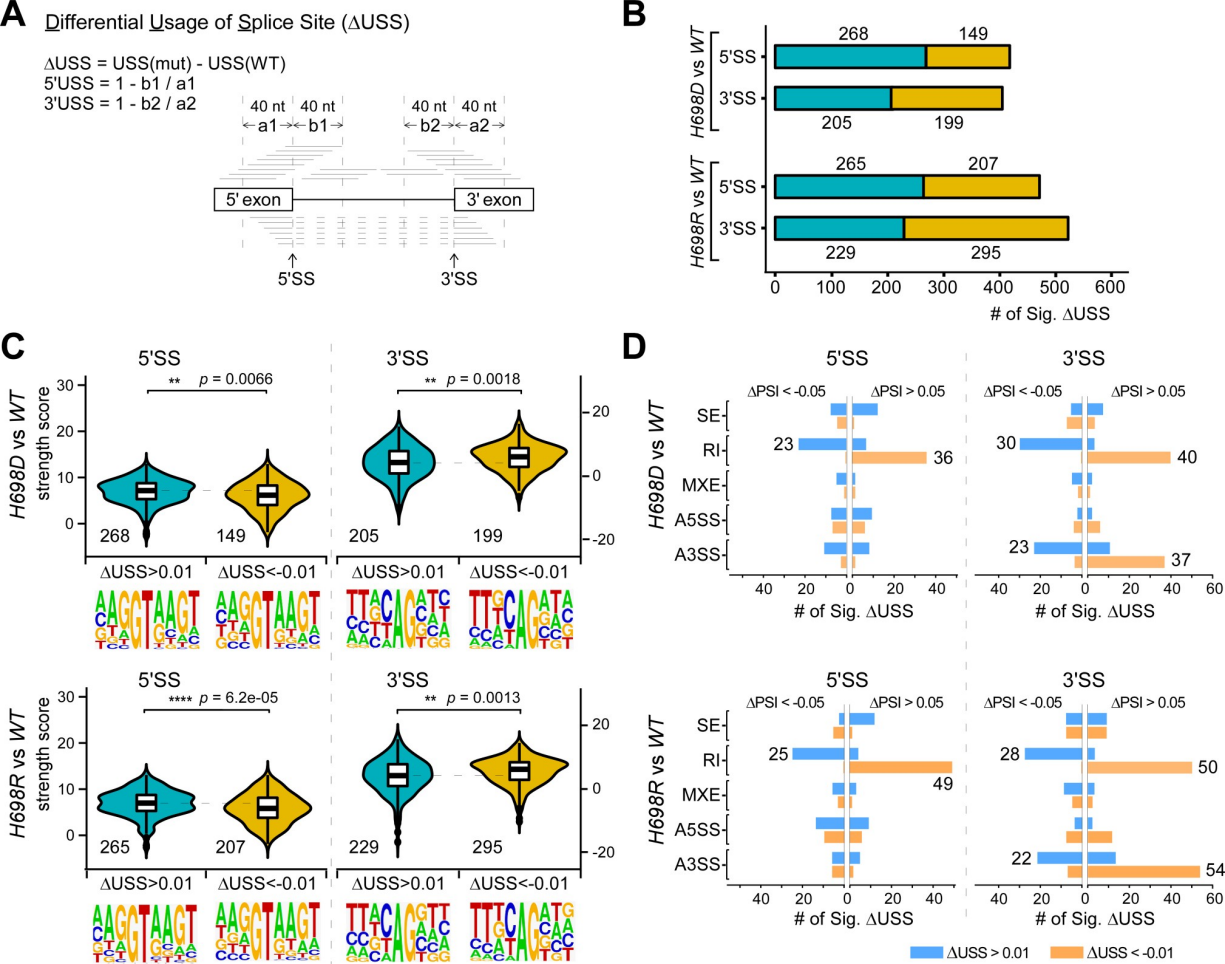
Differential usage of splice sites in the *sf3b1* mutants. (**A**) Schematics for analysis of ΔUSS between fly strains. Transcriptome-wide usages of all *Drosophila* splice sites were individually analyzed by the USS, in which a1 and b1 are coverage of reads that located in the downstream 40 nt of 5′ exons and upstream 40 nt of the 5′ introns, respectively. Similarly, a2 and b2 are coverage of reads that used for USS analysis of 3′ splice sites. (**B**) Significantly changed USSs in the *sf3b1* mutants. The 5′ and 3′SSs with |ΔUSS| > 0.01, FDR < 0.05 were screened in *H698D* and *H698R*. Blue: ΔUSS > 0.01, brown: ΔUSS < -0.01. (**C**) Comparison of splicing signals between SSs with significant changes in usage. The strength of splicing signals was scored by MaxEntScan using the 9-nt sequences of 5′SSs and the 23-nt sequences of 3′SSs [89]. The consensus sequences are visualized by WebLogo [90]. (**D**) AS events from the RI and A3SS groups are enriched in the significantly changed USSs. Left, only the RI-events are enriched in the significantly changed usage of 5′SSs; Right, events from the RI and A3SS groups are enriched in the significantly changed usage of 3′SSs.

Alternative splicing is the consequence of competition/selection between multiple SSs. To address details of SS selection, we used our recently developed tool ΔUSS (Differential Usage of Splice Site) to evaluate all the individual SSs in the *Drosophila* transcriptome [74] (Fig 6A). In total, usages of 417 and 472 of 5′SSs, and 404 and 524 of 3′SSs were significantly changed in *H698D* and *H698R*, respectively (Fig 6B and S6 Table). We further found that the usage-decreased 5′SSs preferentially had weaker splicing signals than the usage-increased 5′SSs and usage-not-changed 5′SSs (|ΔUSS| < 0.01) in both the sf3b1 mutants, exhibiting lower strength scores and relatively less conservation of the last two nucleotides (AG) of the 5′ exon (Figs 6C left and S9); whereas the usage-decreased 3′SSs preferentially had stronger splicing signals than the usage-decreased 3′SSs and usage-not-changed 3′SSs (|ΔUSS| < 0.01), exhibiting stronger strength scores and relatively stronger conservation of the last 3^rd^ nucleotide (C) of the 3′SS (Figs 6C right and S9). These results suggest that a stronger 5′SS could be more easily selected by the two *sf3b1* mutants; vice versa, selection of a stronger 3′SS could be less efficient in both mutants.

We then compared the two data sets of ΔUSS and ΔPSI, and found that the significantly usage-changed 5′SSs were highly enriched only in the group of RI events (Fig 6D left), while the significantly usage-changed 3′SSs were highly enriched in both the RI and A3SS groups (Fig 6D right). These results notably demonstrate that alteration by the *sf3b1-H698* mutants on the 5′SSs preferentially results in intron retention and on the 3′SSs preferentially causes intron retention or alternative 3′SS.

### The *sf3b1-H698R* mutant activates upstream cryptic BSs

**Fig 7:**
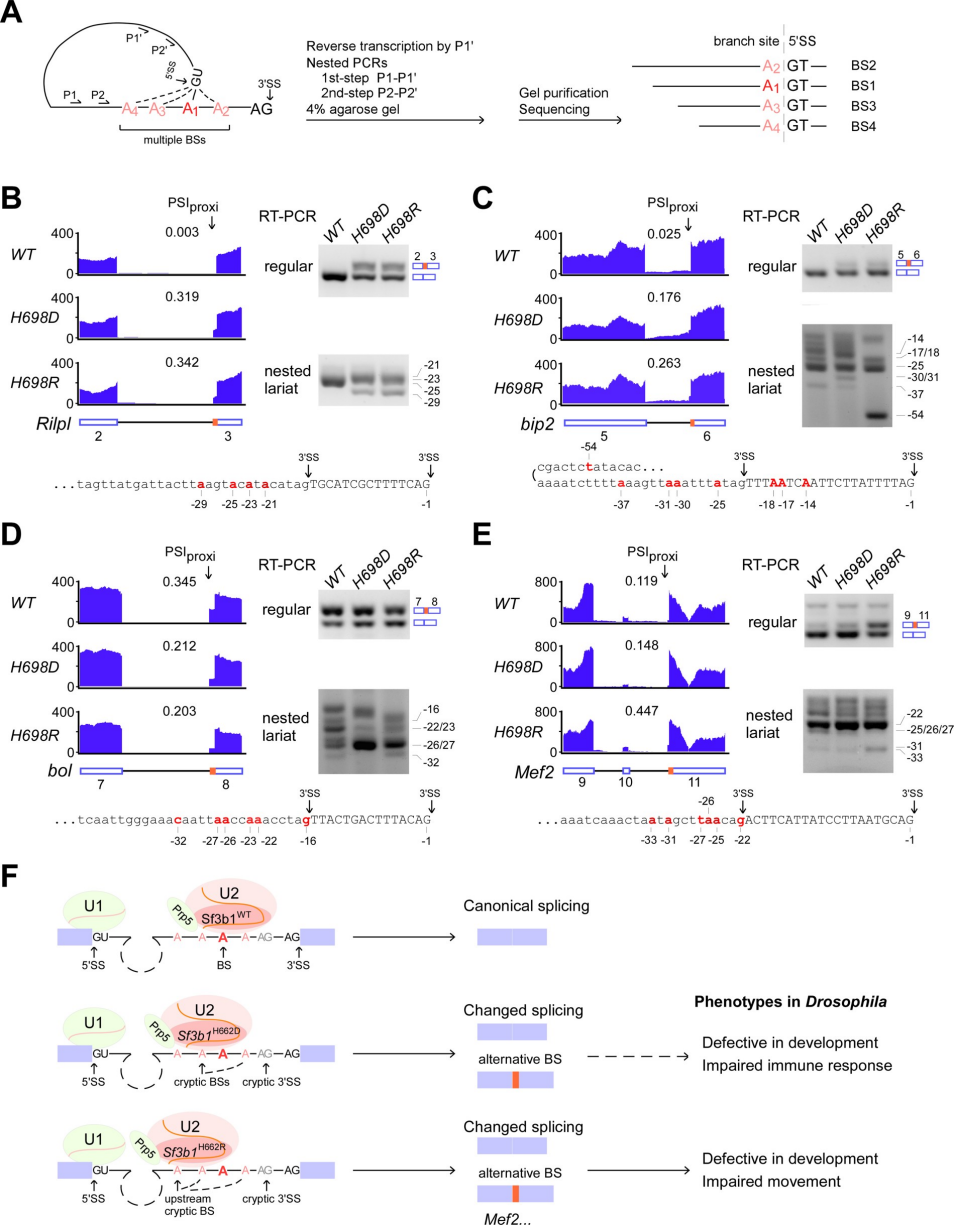
Altered selection of branch sites by *sf3b1* mutants. (**A**) Schematic graph for identification of BS by nested lariat RT-PCR and sequencing. cDNA from the intronic lariat is transcribed using an intron-specific antisense primer P1′, and then amplified by two-steps PCRs using primer sets P1′ + P1 and P2′ + P2. Separated PCR products are cloned for Sanger sequencing, and the multiple used branch sites (BS, red As) are identified according to positions of junctions between the internal intron and upstream 5′SSs. BS selection in the alternatively spliced *Rilpl-*intron 2 (**B**), *bip2-*intron 5 (**C**), *bol*-intron 7 (**D**) and *Mef2*-intron10 (**E**) was identified in the *sf3b1*-*WT* and mutants. The changed A3SS events were validated by regular RT-PCRs, in which PSI values for DS events (red boxes) in each fly strain are indicated. Selected BSs were identified by lariat RT-PCR and Sanger sequencing. (**F**) Schematic model of *sf3b1-H698D* and–*H698R* mutants that alter selection of the intronic branch sites and result in changed AS, and thereby defective *Drosophila* respectively. *Sf3b1*-*H698R* mutant protein enhances the use of upstream cryptic BSs; this is due to its altered SF3B1 conformation with either changed Prp5—Sf3b1 interaction or changed pre-mRNA—Sf3b1 interaction. Multiple branch sites (red As) and alternative splice sites (red boxes) are indicated.

We previously observed opposite effects on splicing of suboptimal BS substrates between the yeast counterpart mutants *Hsh155-H331D* and–*H331R* using the well-established *ACT1-CUP1* reporter system [38]. Here, a large number of AS events were similarly changed in both the mutant flies (Fig 4B). We hypothesized that this might be due to competition between multiple branch sites in the related *Drosophila* introns. To test this hypothesis, we sequenced nested PCR products of the reverse transcribed lariats from four A3SS events to identify BSs used in the *WT* and *sf3b1* mutants (Figs 7A and S10 and details in Materials and Methods).

For example, as confirmed by RT-PCRs, an upstream cryptic 3′SS in intron 2 of *Rilpl* was more used, and, to a similar extent, in both *sf3b1* mutants in comparison to the *WT* (Figs 7B and S7E). Using specific primers, splicing intermediate lariats of the *Rilpl*-intron 2 from the three fly strains were reversely transcribed and amplified through a two-steps nested PCR. After gel purification and Sanger sequencing of dozens of plasmid clones from each strain, we identified four BSs from this intron according to the new sequences of ligation between the BS and the 5′SS where the BS-A is often mutated to T due to reverse transcription of a unique 2′,5′-phosphodiester bond (S10 Fig), only two of them (positions -21 and -23) were used in the *WT* and their selection was greatly decreased in the two *sf3b1* mutants. The *H698R* mutant preferentially used the most upstream BS at the position -29, whereas the *H698D* mutant nearly equally selected three other BSs (Figs 7B and S9A), suggesting a different preference of BS selection by the *Sf3b1-WT*, *-H698D* and *-H698R* proteins. Similarly, multiple selections of BS were also observed in the other two tested shared A3SS events, in which an upstream cryptic 3′SS in intron 5 of *bip2* and a downstream cryptic 3′SS in intron 7 of *bol* was used more in the *sf3b1* mutants than in the *WT* (Figs 7C and 7D, S7D and S7F). Multiple BSs were identified in these two introns by lariat sequencing. For intron 5 of *bip2*, in total eight branch sites were used in the three fly strains, in which the *WT* prefers to use position -25A, the *H698D* mutant prefers to equally use positions -25A, -30A and -31A, and the *H698R* mutant prefers to use the most upstream -54U (Figs 7C and S9B). For the intron 7 of *bol*, in total six branch sites were used in the three fly strains, in which the *WT* prefers to use positions -16G and -26A, the *H698D* mutant prefers to use position -26A, and the *H698R* mutant prefers to -26A and the most upstream -32C (Figs 7D and S9C).

We also tested BS selection in intron 10 of *Mef2*, in which the changed A3SS only occurs in the *H698R* mutant. In total, six branch sites were used in the three fly strains, in which the *WT* prefers to use positions -22G, -25A and -27U, the *H698D* mutant prefers to use positions -22A, and the *H698R* mutant prefers to -22A and the most upstream -33A (Figs 7E and S9D). Taken together, investigation of branch sites selection in those four introns revealed that multiple aberrant branch sites are used in the two *sf3b1-H698* mutants, and the far upstream branch sites are preferentially used by the *H698R* mutant, suggesting a different branch site selection between the two oppositely-charged mutations.

## Discussion

The high frequency of SF3B1 mutations and their progressive stimulation in many cancers reflect the critical roles of SF3B1 protein in the recognition and selection of intronic splice sites and branch sites and thereby accurate gene expression. In this study, using the *Drosophila melanogaster* system, we focused on the hotspot disease mutation site His662 and investigated the effects of its mutations at the levels of both splicing and development.

Structural evidence reveals that SF3B1 has an open-state conformation in the isolated SF3b core [48] and in the 17S U2 snRNP [28], where the super-helical structure of SF3B1 HEATs 1–6 and 9–12 interacts with the N-terminal region of the RNA helicase Prp5. This structural information is consistent with our previous biochemical data in the yeast system [38]. In the cryo-EM structures of B^act^ complex, the SF3B1 HEATs adopt a closed-state conformation of a ring-like structure through which the 3′-end of the intron with the branch site is threaded [55,75,76]. This conformational change from open to closed state is facilitated by the RNA helicase Prp5. However, the influence on these conformational changes by disease mutations in the SF3B1 HEATs is not yet clear.

Differential A3SS events have been found to be the dominant splice changes in other studied SF3B1 mutations, such as K700E, K666 mutations and R625 mutations in human cells lines [40,47,59] and K700E in mouse models [77–79]. Here, we found that many AS events in the other four types of alternative splicing are also changed in the *Drosophila sf3b1-H698* mutants (Fig 4A). This could be due to a combination of two reasons: i) the intronic structures including splicing consensus sequences and average intron length in *Drosophila* are different from those in mammals; ii) the mutated histidine residue may have a different influence on the conformational changes of Sf3b1 during the transition between spliceosomal complexes compared to other HEAT motif mutations. For example, many identified intron retention events in this study are connected with the decreased usage of 5′SSs, and those introns are relatively short, a characteristic of fly introns in comparison to mammalian introns.

Our previous study in yeast found that the *Hsh155*-H331D mutation decreases yeast Hsh155/SF3B1 interaction with Prp5, whereas the *Hsh155*-H331R mutation enhances interaction with Prp5, and they have opposite splicing effects on suboptimal BS region substrates, indicating that selection specificities of the branch sites by these two mutants are different. In this study, we found that there are ~1,000 changed AS events in each *sf3b1-H698* mutant fly. Sequencing of lariat products from four changed A3SS events, we demonstrate that aberrant branch sites are used in the two *sf3b1-H698* mutants, of which the *H698R* mutant prefers to use far upstream cryptic branch sites, showing a different characteristic from the *H698D* mutant. The human H662 residue of SF3B1 directly interacts with the intronic 13^th^-15^th^ nucleotides upstream of the branch site adenosine in the Cryo-EM structure of B^act^ complex [55] (S11 Fig). Therefore, we propose that the *Drosophila* His698 residue mutation to a stronger positively-charged Arg residue results in either a disordered open-state conformation of Sf3b1 that is defective in Sf3b1—Prp5 interaction, or a less stable close-state conformation that alters the pre-mRNA—Sf3b1 interaction (Fig 7F). Together, the aberrant selection of BS by the two *sf3b1* mutations demonstrates that this conserved Histidine residue in Sf3b1 contributes to splicing proofreading at the intronic branch site region.

Although these two oppositely-charged mutations cause many similar defects during the *Drosophila* developmental stages, *H698D* and *H698R* mutant flies also have different defects, such as innate immune response and movement (Fig 7F). We find that the *H698R* mutant specifically alters splicing of many muscle and neuron-related genes, whereas the *H698D* mutant changes expression of several immune response genes. Expression of the RB isoform of *Mef2*, which is downregulated in the *sf3b1-H698R* mutant, partially rescues the climbing defects caused by the *H698R* mutation (Fig 7F).

As mentioned above, previous studies had showed that other *sf3b1* mutations resulted in alternative 3′SS and non-canonical branch site selection. In this study, we provide data of two H698 oppositely-charged mutations in *Drosophila*; they have different effects on splicing of a variety of genes, as well as exhibiting different phenotypes. We reveal that far upstream branch sites are used by the *H698R* mutant, but not by the *H698D* mutant. These novel findings in *Drosophila* suggest that mutations at the same residue of SF3B1 in cancers would be mechanistically different in changes of alternative splicing on different substrate genes, and thus would be predicted to have different progression during the development of cancers.

It has been reported that splicing factor mutations in SRSF2 and U2AF1 result in enhanced R-loops and thereby impaired transcription [80]. Therefore, a portion of those expression-changed genes in the *sf3b1-H698* mutants could be directly caused by altered transcription. In addition, the variety of developmental defects found in this study allow us to expect more SF3B1 mutations to be found in other human diseases.

## Materials and methods

### Fly strains and culture

The wild type (*WT*) *Drosophila melanogaster* used in this study is a *w1118* isogenic strain (BDSC 5905). Point mutant strains were constructed using the CRISPR/Cas9 system [74]. In brief, the target sequence of each guide RNA (sgRNA) was selected, donor plasmids with point mutations and the adjacent 3 kb sequences as homologous arms were constructed using pMD18-T (Fig 1B), and the gRNA and donor plasmids were co-injected into embryos of the transgenic line *nanos-Cas9* by UniHuaii Technology Company. Specific primers that distinguished point mutations were used for genomic PCRs to screen for the desired alleles, which were further validated by Sanger sequencing of amplicons. The flies obtained were then crossed for at least five generations with the *WT* strain to eliminate potential off-target events. Homozygous point mutant flies were maintained and cultured on standard cornmeal agar medium. All primers and oligos used are listed in S7 Table.

*Mef2-GAL4* on the 3rd Chr. was a gift from Prof. Dong Yan at CEMPS, transgenic *UAS-Mef2(RB)* strain was constructed through incorporating pUAS-T(CDS of *Mef2-RB*) at attP2 site on the 3rd Chr. Then *sf3b1-H698* mutant strains were separately crossed with *Mef2-GAL4* and *UAS-Mef2(RB)*, and the strains of *sf3b1-H698R/H698R;Mef2-GAL4/UAS-Mef2(RB)* and s*f3b1-H698D/H698D;Mef2-GAL4/UAS-Mef2(RB)* were finally obtained by further crossing.

### Western blot and immunohistochemistry

Western blot signals of Sf3b1 and Tubulin were detected using Rabbit anti-Sf3b1 antibody (antigen: VDEDEDDGFPVPQKRT) and anti-tubulin antibody (Sigma), respectively. Fat bodies of third-instar larvae were dissected, followed by incubation with the primary antibody Rabbit anti-Sf3b1 (1:500) and then the secondary antibody goat anti-rabbit Alexa Fluor 594. DAPI (1:2000; Sigma) was used for staining the nuclei. Images were acquired using a Carl Zeiss LSM880 confocal microscope.

### Fecundity and hatching assays

The number of eggs laid per female fly was measured as described [81]. Briefly, ten individual female adults (16–20_hr) from each strain were passed to new vials, and their eggs laid per vial were counted at each time point. Four sets of 300 eggs from each strain were collected and counted for hatching rates under standard condition [82]. Statistical differences were determined according to t-tests. All statistical analyses were performed with GraphPad Prism 7 (San Diego).

### Time of developmental stages

Homozygous flies were mated and their laid eggs were collected in a 0.5_hr window and counted as time zero. The 1st instar larvae (270 for each strain) were picked and transferred into new vials with standard food at the 30_hrs post-laying. Pupation and eclosion of flies were counted in regular intervals [82]. Their lifespans were measured as described [81]. Briefly, 200 virgin females and 200 virgin males were maintained in vials at a density of 25 flies per vial on standard food. Flies were transferred to new vials every 2–3 days and the dead flies were counted, and the survival median time was analyzed using GraphPad Prism curves.

### Climbing assay

Climbing ability (negative geotaxis) was measured as described [83]. Ten flies in a vial, three vials of adults at the 5th, 15th, 25th, and 35th days were collected for each strain per assay. Fly climbing was monitored and recorded three times after tapping them to the bottom of the vials, and the height was scored from the photo taken after 6 seconds using RflyDetection software. Multiple climbing flies were processed by Prism. Using a t-test, statistical analyses were presented as mean ± SEM (* p < 0.05, ** p < 0.01, *** p < 0.001). The flight and jump muscle of *Drosophila* were dissected and obtained as described [84].

### Fungal infections

Fungal infection was carried out with 10^7^ spores/ml of *Metarhizium anisopliae* ARSEF 23 (Ma23). Briefly, after gentle shaking to evenly distribute the spores after bathing, 150 flies per sample were moved into fresh vials with food. Non-infection controls were given the same treatment without fungus. Flies were then kept at 29°C and transferred to new vials every two days with counting of the surviving flies. Percentages of survivals were presented by the averages with standard errors, and the median survival days were calculated using GraphPad Prism [85].

### RNA-seq and bioinformatics

Total RNAs from the 5d_adults were isolated by TRIzol (Ambion) and treated with RNase-free DNase I (Invitrogen). Construction of cDNA libraries and sequencing were performed using Illumina HiseqXten-PE150 by Novogene. Raw reads from RNA-seq were quality filtered and trimmed, and then mapped to the *Drosophila melanogaster* genome (dm6) by Hisat [86] and counted by HTseq [87].

Analysis of differentially expressed genes was performed by DEseq2, genes with fold changes > 1.5 and FDR < 0.05 in both two lines were screened as significant. The Gene Ontology (GO) enrichment is analyzed by online DAVID. Differentially spliced (DS) events were analyzed by rMATS [88]. Significant DS events were screened by conditions |ΔPSI| > 0.05 and FDR < 0.05. Differential splice site usage was analyzed by ΔUSS, which is modified from an Unused Index as described [74]. Significant ΔUSS were screened by conditions |ΔUSS| > 0.01, p value <0.05. Muscle-related genes were selected from a list as described [67] and the neural-related genes were selected from a list as described [66].

### RT-PCR, qPCR and lariat RT-PCR

For the regular RT-PCR, reverse transcription was performed using RevertAid Reverse Transcriptase (Thermo), and the cDNA was amplified by Ex-Taq (TaKaRa). qPCR was carried out using SYBR Green Master Mix (Applied Biosystems) in biological triplicates followed by *ΔΔCt* analysis.

The muscle samples for RT-PCR were dissected as described [84] with modifications. Briefly, fly adults were anesthetized and placed in the iced PBS buffer with 0.01% of tween 20, and the head, abdomen and wings were carefully removed. To prepare IFM, two cuts were made to split the thorax open, one was through the ventral cuticle between the two sets of legs and the other was through the dorsal cuticle. The dorsal cut was made off to one side to ensure that the opposite side’s set of DLM fibers are not damaged. The exposed IFM was then picked and placed into a tube with 100 μl PBST and maximum 6 individuals. The dorsal TDT was clamped and stretched slightly, and the jump muscle was freed from the cuticle by cutting the middle leg at the coxa and pleura junction. The TDT sample was then picked and placed into a tube with 100 μl PBST and maximum 6 individuals. Since the muscles cannot be remained in PBST for long time, the whole procedure was performed in less than 20 min, and samples were quickly stored in -70°C freezer. At least 30 flies per strain were dissected to ensure a sufficient number of individuals for further RNA isolation.

For the lariat RT-PCR, cDNA was synthesized by SuperScript IV Reverse Transcriptase (Invitrogen) using an intron-specific antisense primer P1′ (positions see Fig 7A) located close to the 5′-end of an intron. The 1^st^-step PCR was performed using primers P1′ and P1 in 25 μl for 30 cycles, and the 2nd-step PCR was carried out with primers P2′ and P2 in 50 μl after adding 0.2 μl product from the 1st-step for 35 cycles. One-fourth (12.5 μl) of the PCR products were fully separated by 4% agarose for visualization of bands; the remaining PCR products were running on 4% agarose for short time, gel purified by an Axygen kit and then subcloned into a T-vector (Takara). To identify branch sites and their usage frequencies, 10–12 subclones from each strain were picked for Sanger sequencing, and their sequences were aligned according to the junctions between 5′SSs and BSs. All the primers used are listed in S7 Table.

## Supporting information

S1 Fig Construction of *sf3b1-H698D* and *-H698R* mutant strains. (**A**) The mutant strains were screened and validated by genomic PCR and sequencing. Allele specific primers were used for the *WT*, *H698D* and *H698R* strains respectively, while common primers were used for Sanger sequencing. (**B**) mRNA and protein levels of Sf3b1 were not considerably changed in the two mutant strains. Left, RT-PCR for detection of *sf3b1* mRNAs; right, western blot for detection of Sf3b1 protein. (**C**) Cellular location of *Sf3b1* mutations is similar to the WT protein. Cells from the fat body were used for immunohistochemistry. DAPI (blue) defines the region of the nucleus, Sf3b1 was visualized by Alex-594 (red). Images in panels B and C are representatives from multiple assays.
(TIF)

S2 Fig The *sf3b1-698* mutants are defective in fecundity and lifespan. (**A**) Fewer eggs were laid in the early stage by *sf3b1-H689D* and *-H698R* mutants. The laid-eggs were counted from females crossed with males from their own strains. (**B**) Decreased egg-laying of *sf3b1* mutants. In comparison to the WT, females of the two *sf3b1* mutants laid significantly fewer eggs during the first half of test time. Means: WT = 274.1, H698D = 186.1, p = 0.0015, H698R = 182.3, p = 0.0002. Females of the two sf3b1 mutants laid no significantly changed eggs during the late half of test time. Means: WT = 175, H698D = 195.5, p = 0.5296, H698R = 173.4, p = 0.9437. Data represent the mean ± SEM from ten samples from each strain. (**C**) Shorter lifespans of the *sf3b1* mutants. Median survival days were measured and listed. Median survival: WT = 72, D = 58, p < 0.0001 (****), R = 58, p < 0.0001 (****).
(TIF)

S3 Fig The two *Drosophila* lines of each mutant are highly consistent. (**A**) Correlation analysis of overall transcriptomic genes expression between the two lines of each *sf3b1* mutants. (**B**) Correlation analysis of differentially expressed genes’ log_2_FoldChange between the two lines of each mutant.
(TIF)

S4 Fig Total numbers of DS events in the two *sf3b1-H698* mutants. For each mutant, five types of events are shown. Bar in light colors, ΔPSI decreased events; bar in dark colors, ΔPSI increased events.
(TIF)

S5 Fig Validation of AS changes in the two sf3b1 mutant strains by RT-PCR. AS events were randomly picked from results of the rMATs analysis, and RT-PCR were performed multiple times from biological samples.
(TIF)

S6 Fig Scatter plots of significantly changed AS events from genes that are involved in neural development in the *sf3b1-H698* mutants. Orange dots, events in *H698D*; green dots: events in *H698R*; black dots with gene labeling: unique events in *H698R*.
(TIF)

S7 Fig Sashimi plots of six alternative splicing changed genes in the *WT* and *sf3b1-H698* mutant strains. Data of the *WT* strain are shown in red, *sf3b1-H698D* in blue, and *sf3b1-H698R* in green. Numbers of all the exon-exon junction reads are indicated, and the AS changed exons or introns are shown in red at the bottom of each panel.
(TIF)

S8 Fig Alternative splicing isoforms of the *Mef2* gene in *Drosophila*. Eleven isoforms of *Mef2* are obtained from Flybase and confirmed by our RNA-seq data. The isoforms are named by Flybase and we number the exons considering both clarity and consistency. Blue rectangles: CDS exons, blue boxes: UTRs, and number of amino acids from each isoform-coded protein, if expressed, are also listed. Major changed isoforms in the *sf3b1-H698R* strain are indicated.
(TIF)

S9 Fig Significantly changed USSs in the sf3b1 mutants. The 5′ and 3′SSs with |ΔUSS| > 0.01, FDR < 0.05 were screened in *H698D* and *H698R* and compared with the usage-not-changed SSs (|ΔUSS| < 0.01). Blue: ΔUSS > 0.01, brown: ΔUSS < -0.01, grey: |ΔUSS| < 0.01. Values of mean, median, and SE from each group are presented.
(TIF)

S10 Fig Identification of used branch sites and their frequencies in the *WT* and *sf3b1-H698* mutant strains. PCR products from the amplified lariats of *Rilpl*-intron 2 (**A**), *bip2*-intron 5 (**B**), *bol*-intron 7 (**C**) and *Mef2*-intron 10 (**D**) were sequenced and aligned, and the usage frequencies of BSs were calculated for each strain.
(TIF)

S11 Fig The H662 residue of SF3B1 interacts with the pre-mRNA in the activated spliceosomal B^act^ complex. (**A**) SF3B1 interacts with the BS—U2 snRNA duplex. (**B**) A zoom on the region around the H662 residue that interacts with the upstream of BS. Cyan, SF3B1; orange, pre-mRNA; violet, U2 snRNA; yellow sticks, H662 residue; red, BS-A (branch site adenosine); numbers, nucleotide positions of the pre-mRNA from the BS to upstream. This figure is rendered from PDB ID: 5Z56 (Zhang *et al*., Cell Res 2018) using PyMOL.
(TIF)

S1 Table Summary of SF3B1 mutations in human cancers. (DOCX)

S2 Table RNA-seq samples and reads in this study. (DOCX)

S3 Table Significantly changed gene expression in the *sf3b1* mutants. (XLSX)

S4 Table Significantly changed AS events in the *sf3b1* mutants. (XLSX)

S5 Table Significantly changed AS events in the muscle and neural-related genes in the *sf3b1* mutants. (XLS)

S6 Table Significantly changed USS in the *sf3b1* mutants. (XLSX)

S7 Table Primers used in this study. (DOCX)
