# Supplementary figures and images for: Two oppositely-charged sf3b1 mutations cause defective development, impaired immune response, and aberrant selection of intronic branch sites in Drosophila
Source: PLoS Genet. 2021 Nov 1;17(11):e1009861. doi: 10.1371/journal.pgen.1009861 (PMC8559932; doi:10.1371/journal.pgen.1009861)

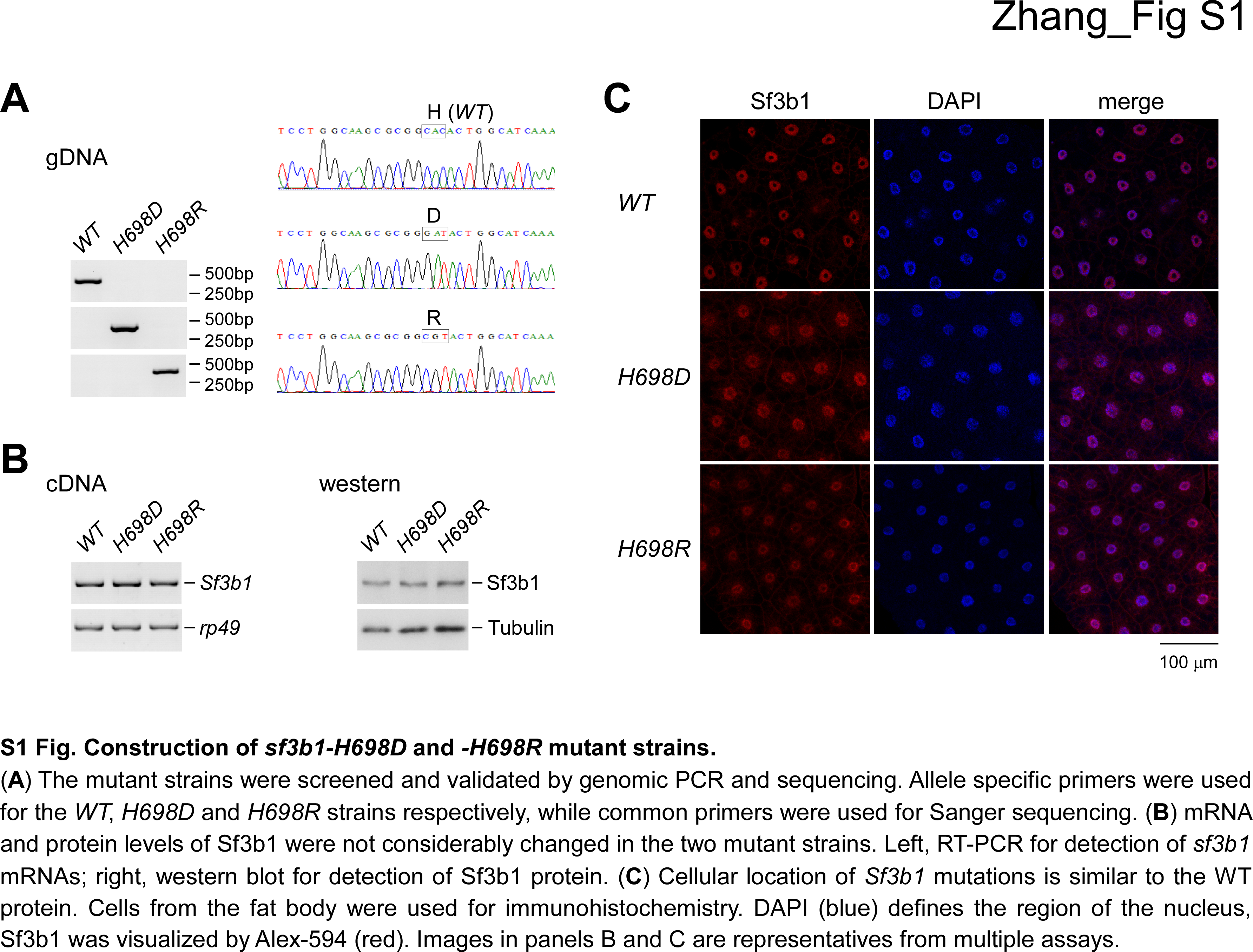

Supplement: S1 Fig — (A) The mutant strains were screened and validated by genomic PCR and sequencing. Allele specific primers were used for the WT, H698D and H698R strains respectively, while common primers were used for Sanger sequencing. (B) mRNA and protein levels of Sf3b1 were not considerably changed in the two mutant strains. Left, RT-PCR for detection of sf3b1 mRNAs; right, western blot for detection of Sf3b1 protein. (C) Cellular location of Sf3b1 mutations is similar to the WT protein. Cells from the fat body were used for immunohistochemistry. DAPI (blue) defines the region of the nucleus, Sf3b1 was visualized by Alex-594 (red). Images in panels B and C are representatives from multiple assays. (TIF) [file pgen.1009861.s001.tif]

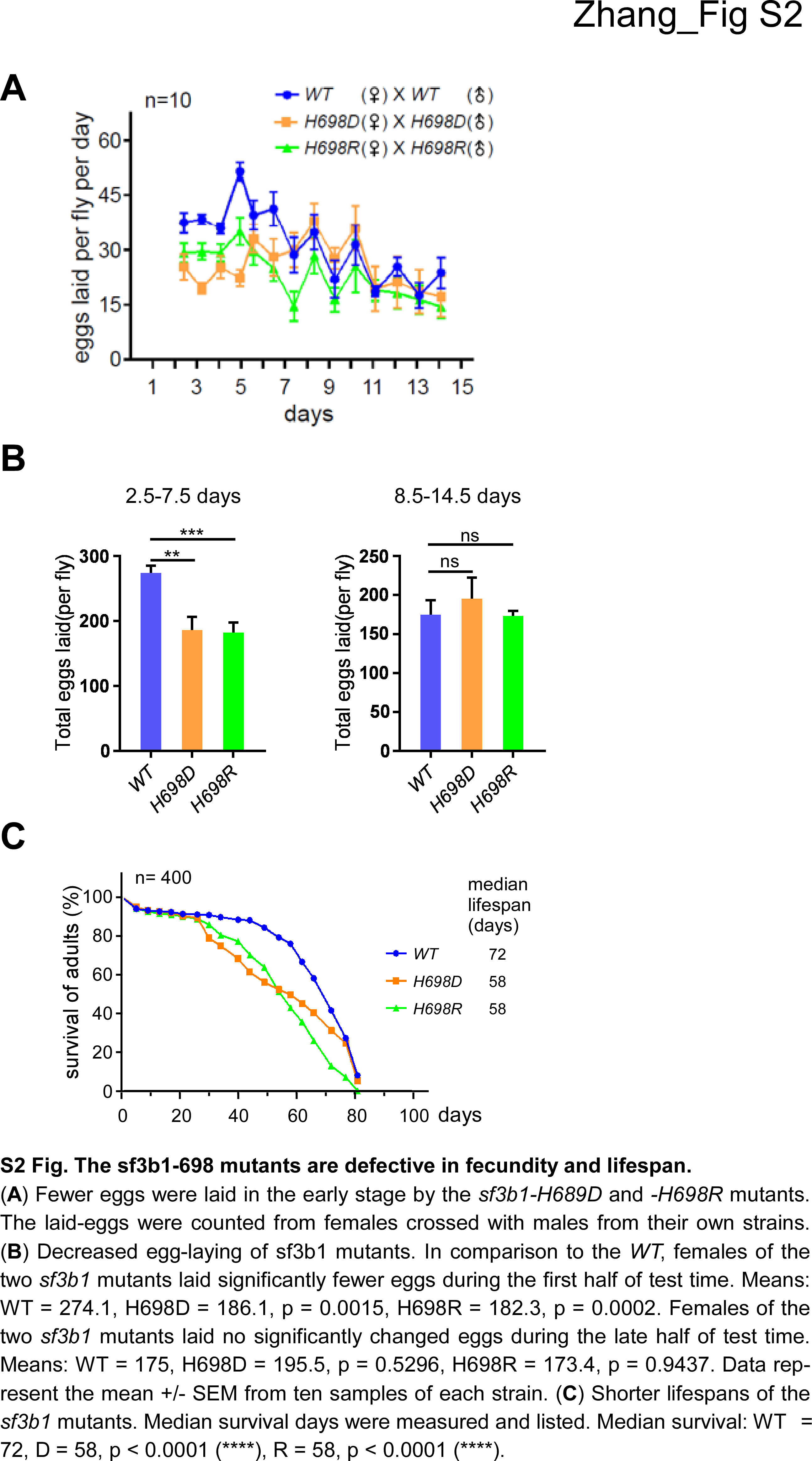

Supplement: S2 Fig — (A) Fewer eggs were laid in the early stage by sf3b1-H689D and -H698R mutants. The laid-eggs were counted from females crossed with males from their own strains. (B) Decreased egg-laying of sf3b1 mutants. In comparison to the WT, females of the two sf3b1 mutants laid significantly fewer eggs during the first half of test time. Means: WT = 274.1, H698D = 186.1, p = 0.0015, H698R = 182.3, p = 0.0002. Females of the two sf3b1 mutants laid no significantly changed eggs during the late half of test time. Means: WT = 175, H698D = 195.5, p = 0.5296, H698R = 173.4, p = 0.9437. Data represent the mean ± SEM from ten samples from each strain. (C) Shorter lifespans of the sf3b1 mutants. Median survival days were measured and listed. Median survival: WT = 72, D = 58, p < 0.0001 (****), R = 58, p < 0.0001 (****). (TIF) [file pgen.1009861.s002.tif]

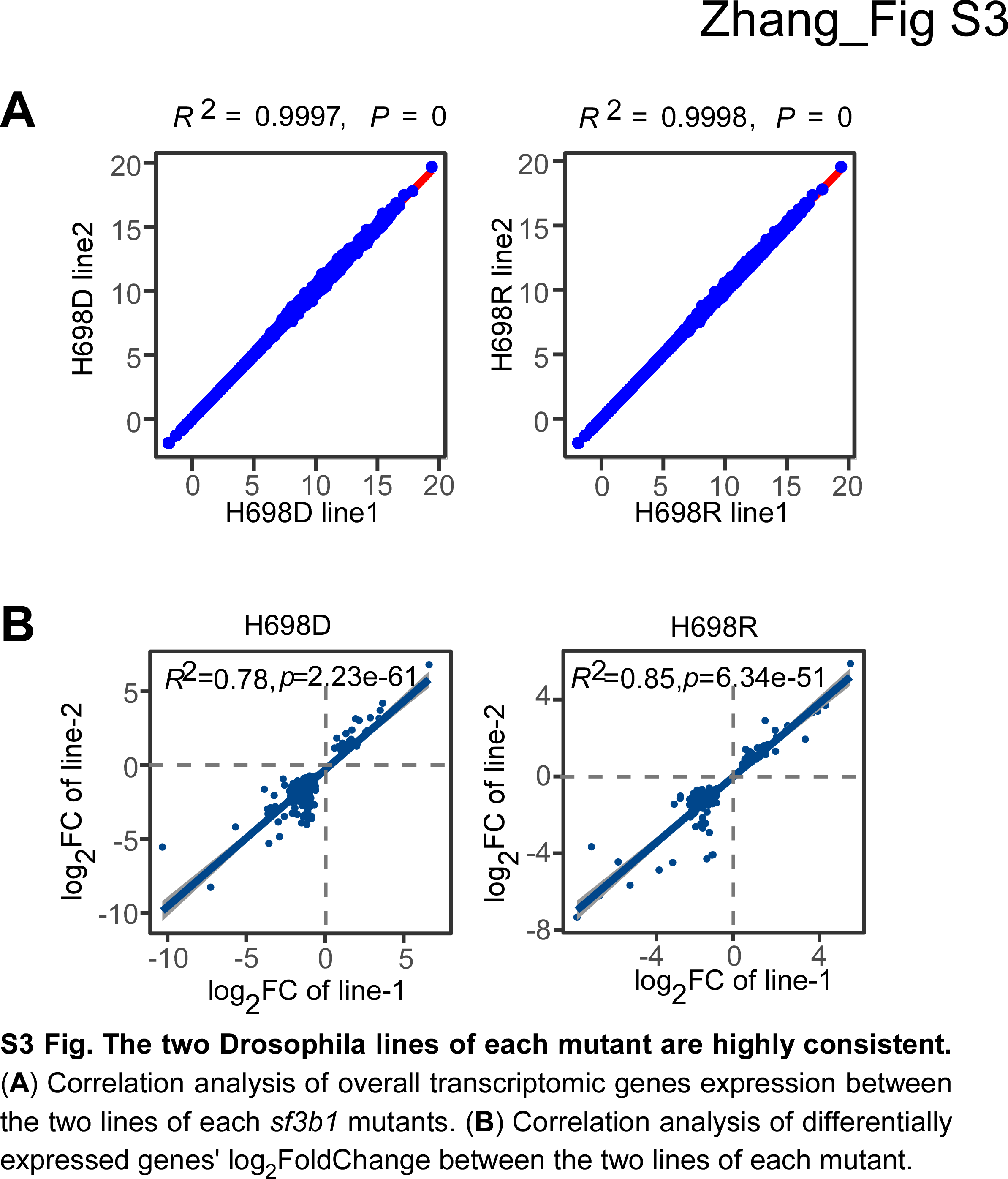

Supplement: S3 Fig — (A) Correlation analysis of overall transcriptomic genes expression between the two lines of each sf3b1 mutants. (B) Correlation analysis of differentially expressed genes’ log2FoldChange between the two lines of each mutant. (TIF) [file pgen.1009861.s003.tif]

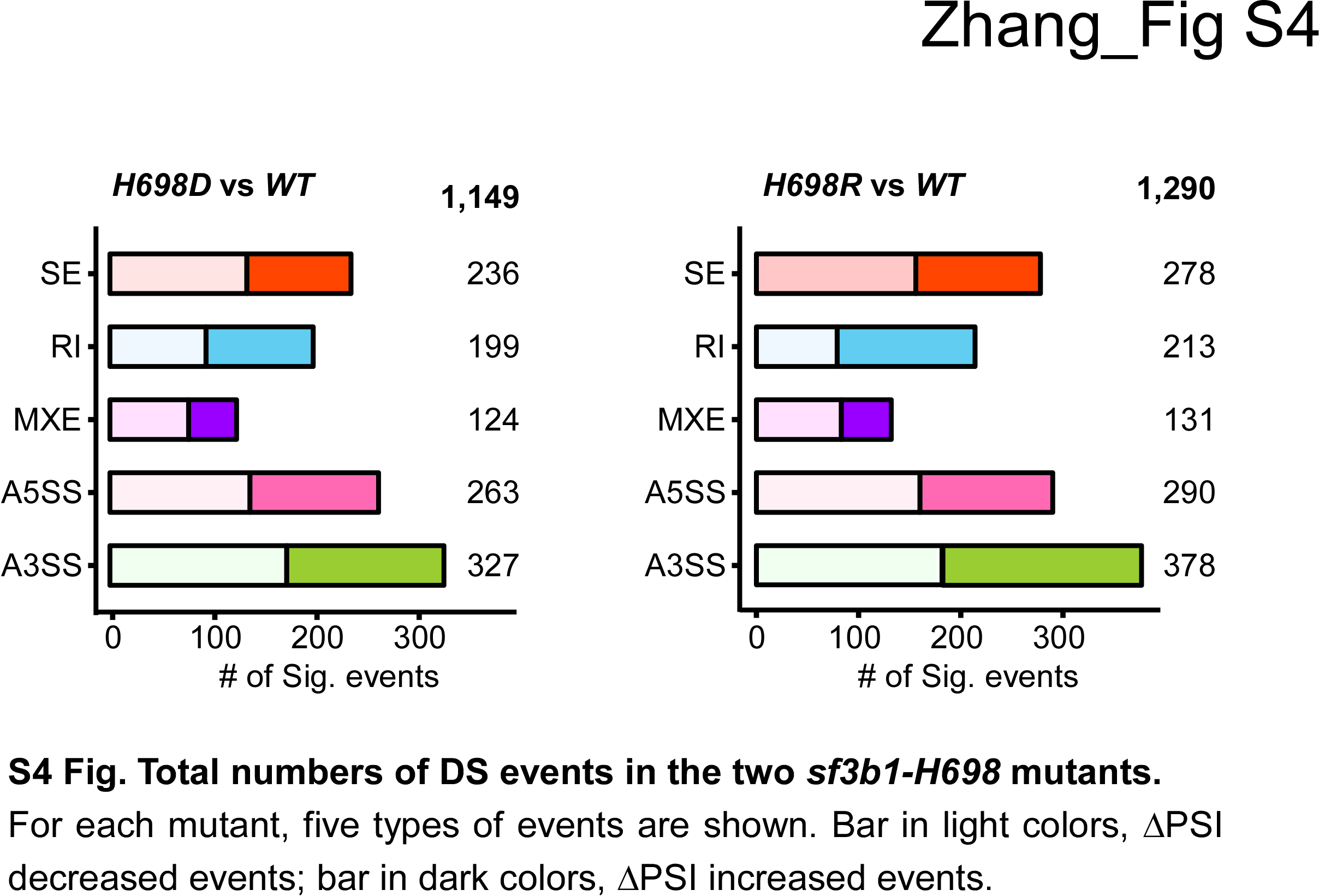

Supplement: S4 Fig — For each mutant, five types of events are shown. Bar in light colors, ΔPSI decreased events; bar in dark colors, ΔPSI increased events. (TIF) [file pgen.1009861.s004.tif]

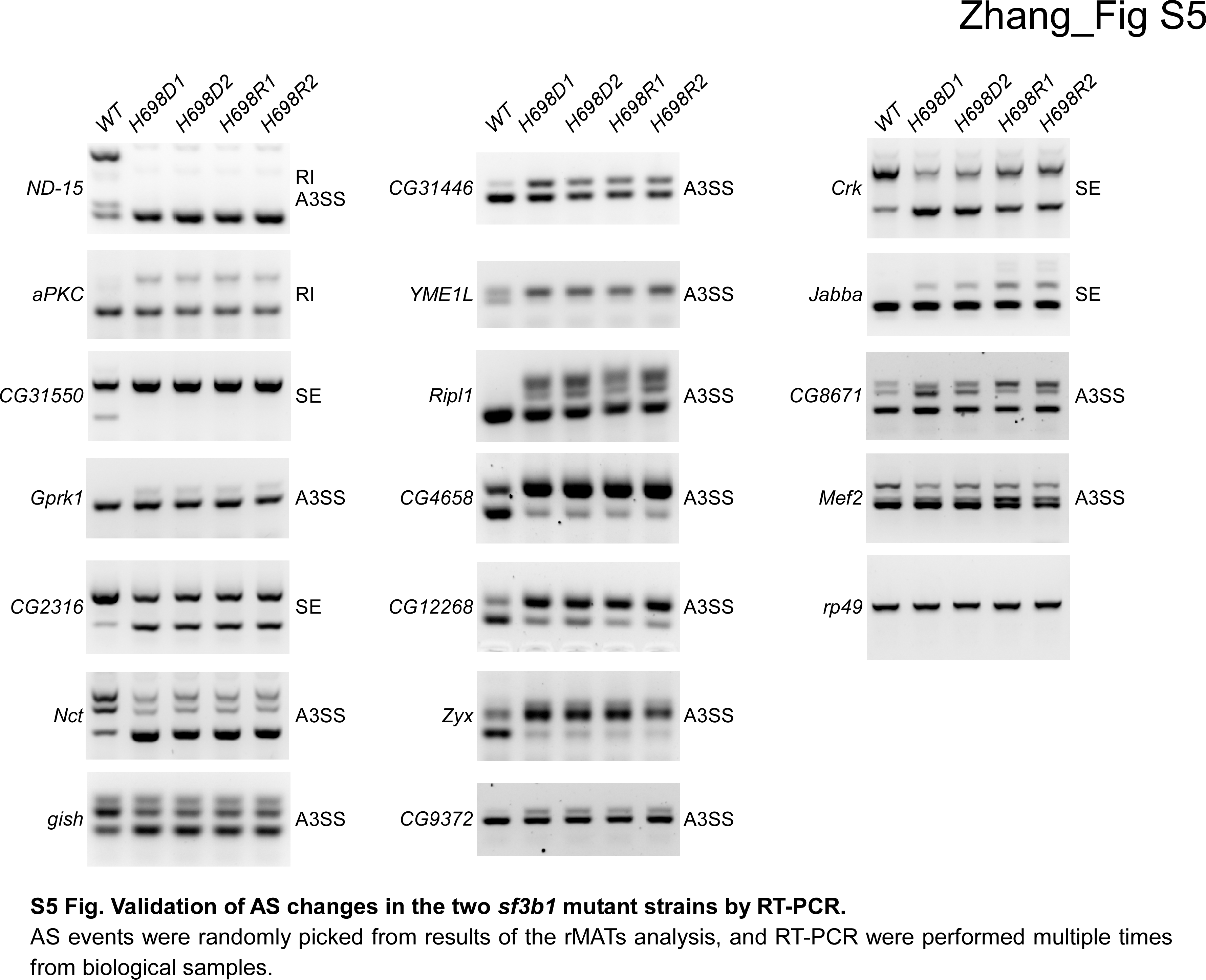

Supplement: S5 Fig — AS events were randomly picked from results of the rMATs analysis, and RT-PCR were performed multiple times from biological samples. (TIF) [file pgen.1009861.s005.tif]

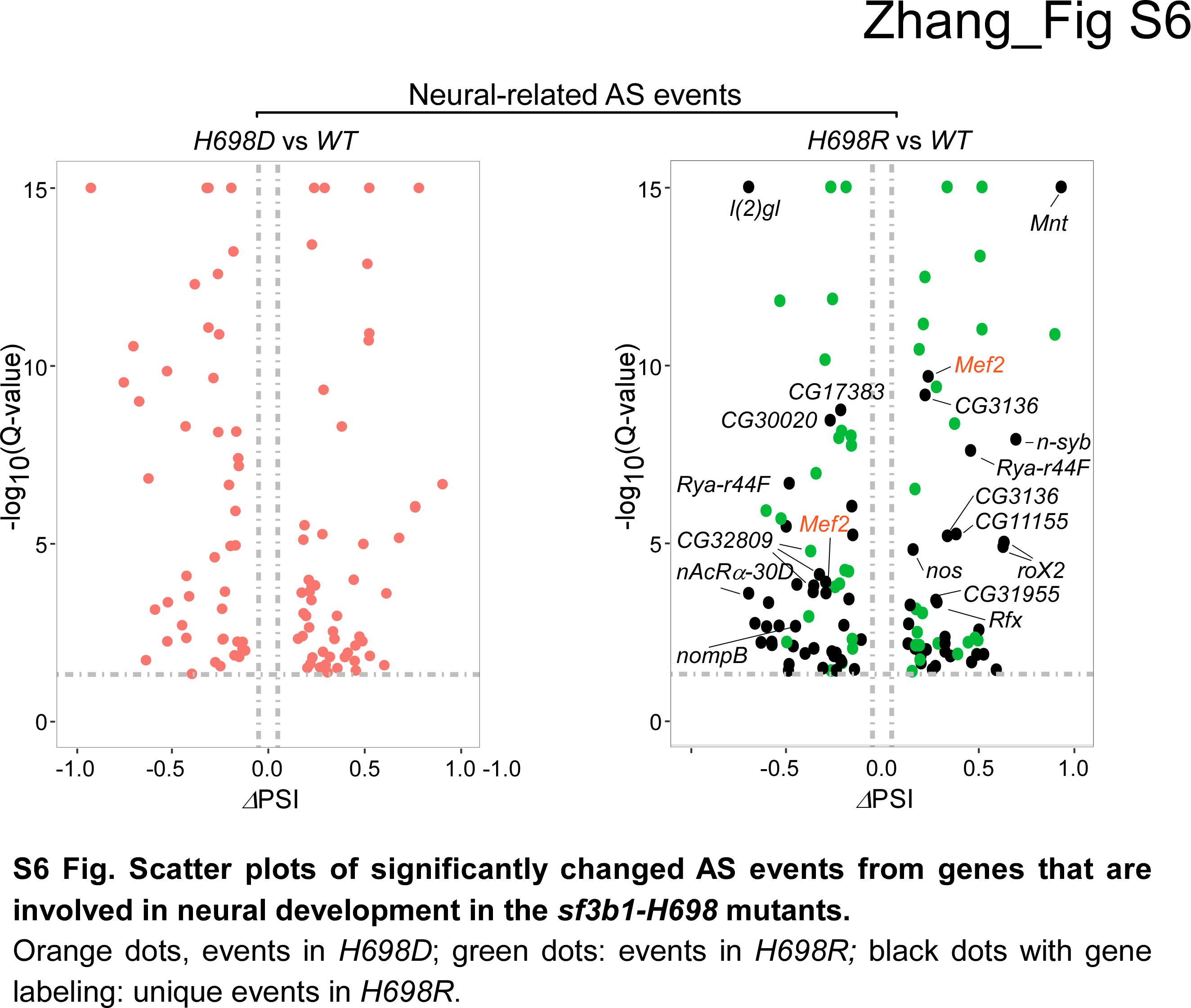

Supplement: S6 Fig — Orange dots, events in H698D; green dots: events in H698R; black dots with gene labeling: unique events in H698R. (TIF) [file pgen.1009861.s006.tif]

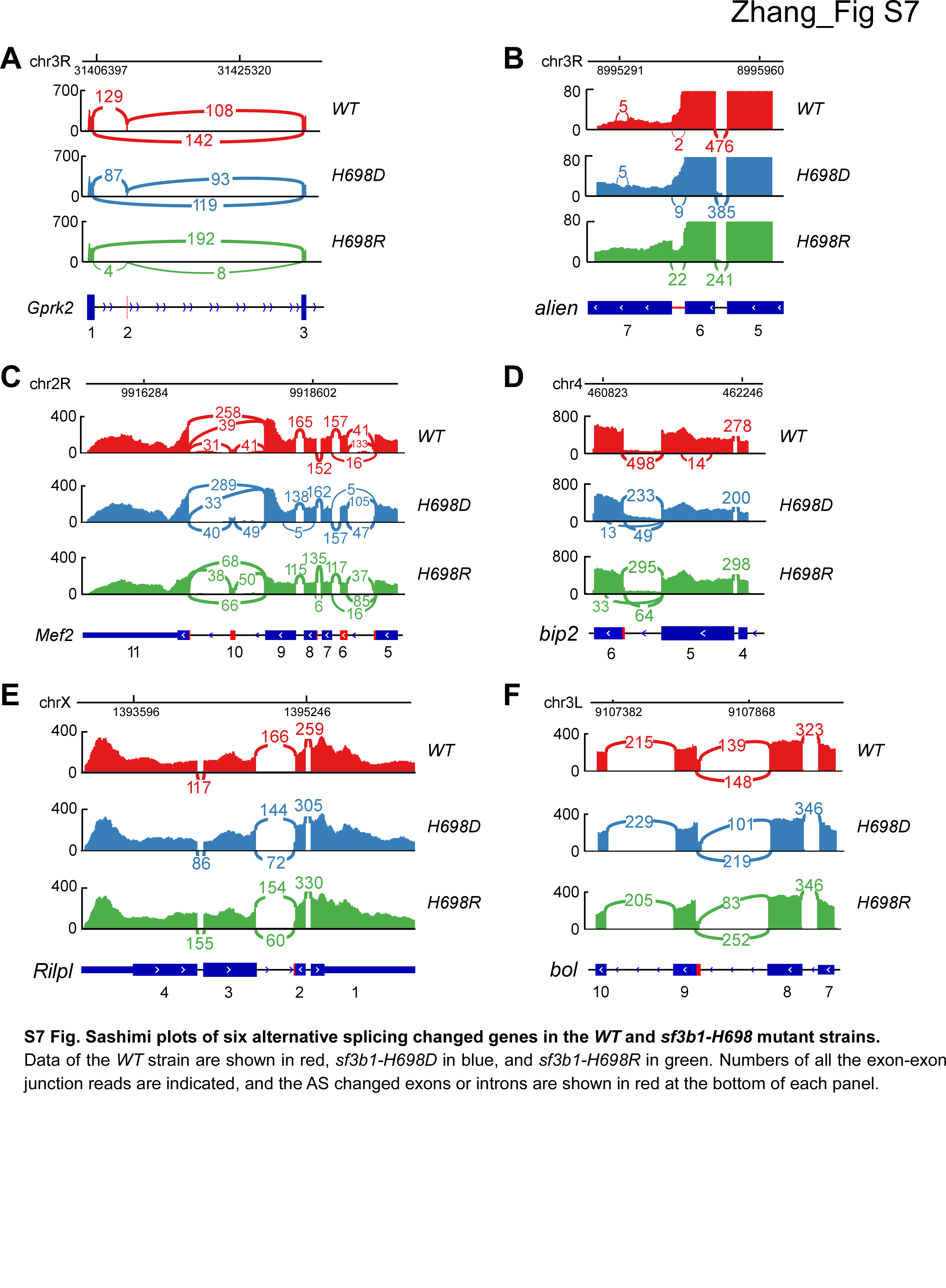

Supplement: S7 Fig — Data of the WT strain are shown in red, sf3b1-H698D in blue, and sf3b1-H698R in green. Numbers of all the exon-exon junction reads are indicated, and the AS changed exons or introns are shown in red at the bottom of each panel. (TIF) [file pgen.1009861.s007.tif]

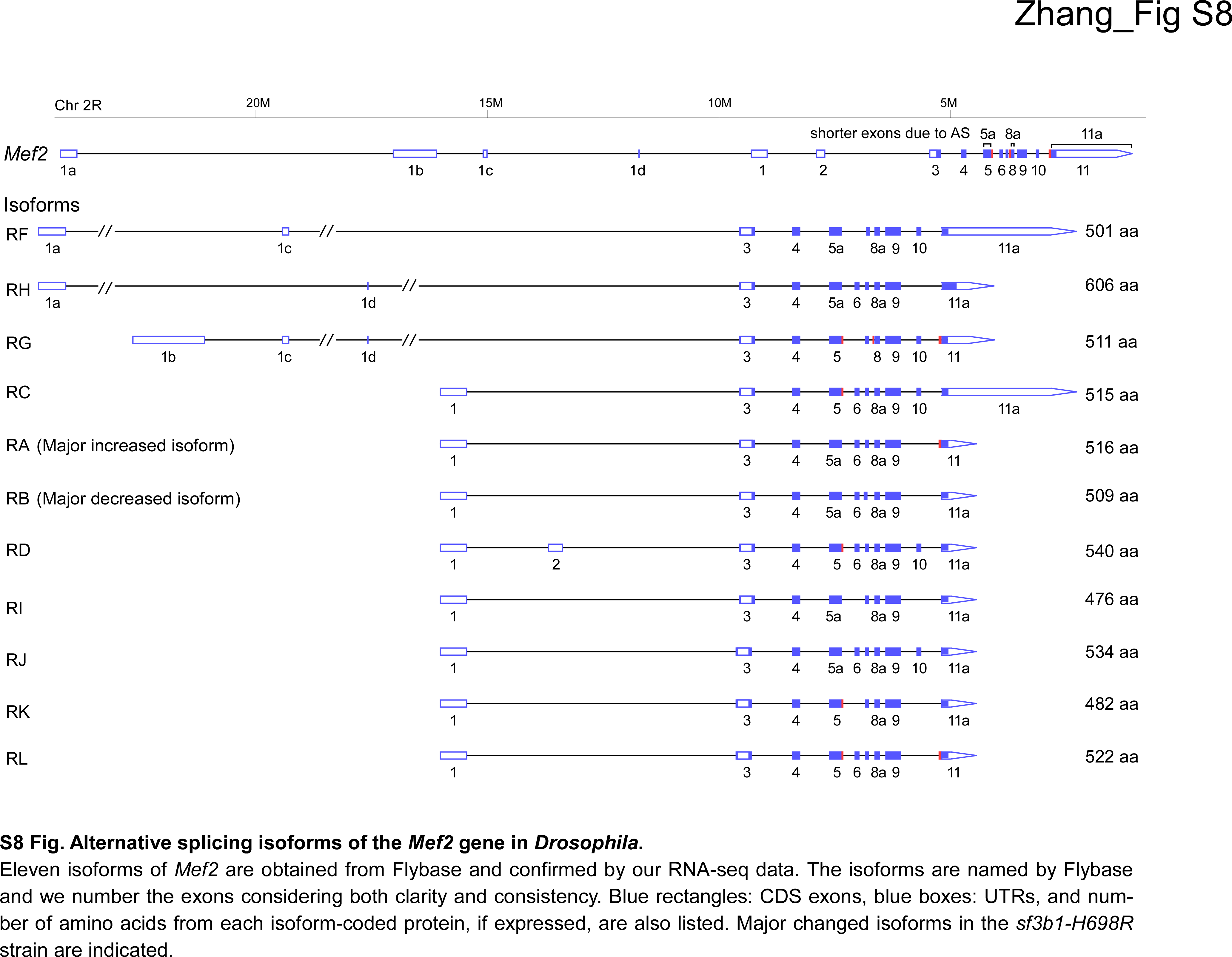

Supplement: S8 Fig — Eleven isoforms of Mef2 are obtained from Flybase and confirmed by our RNA-seq data. The isoforms are named by Flybase and we number the exons considering both clarity and consistency. Blue rectangles: CDS exons, blue boxes: UTRs, and number of amino acids from each isoform-coded protein, if expressed, are also listed. Major changed isoforms in the sf3b1-H698R strain are indicated. (TIF) [file pgen.1009861.s008.tif]

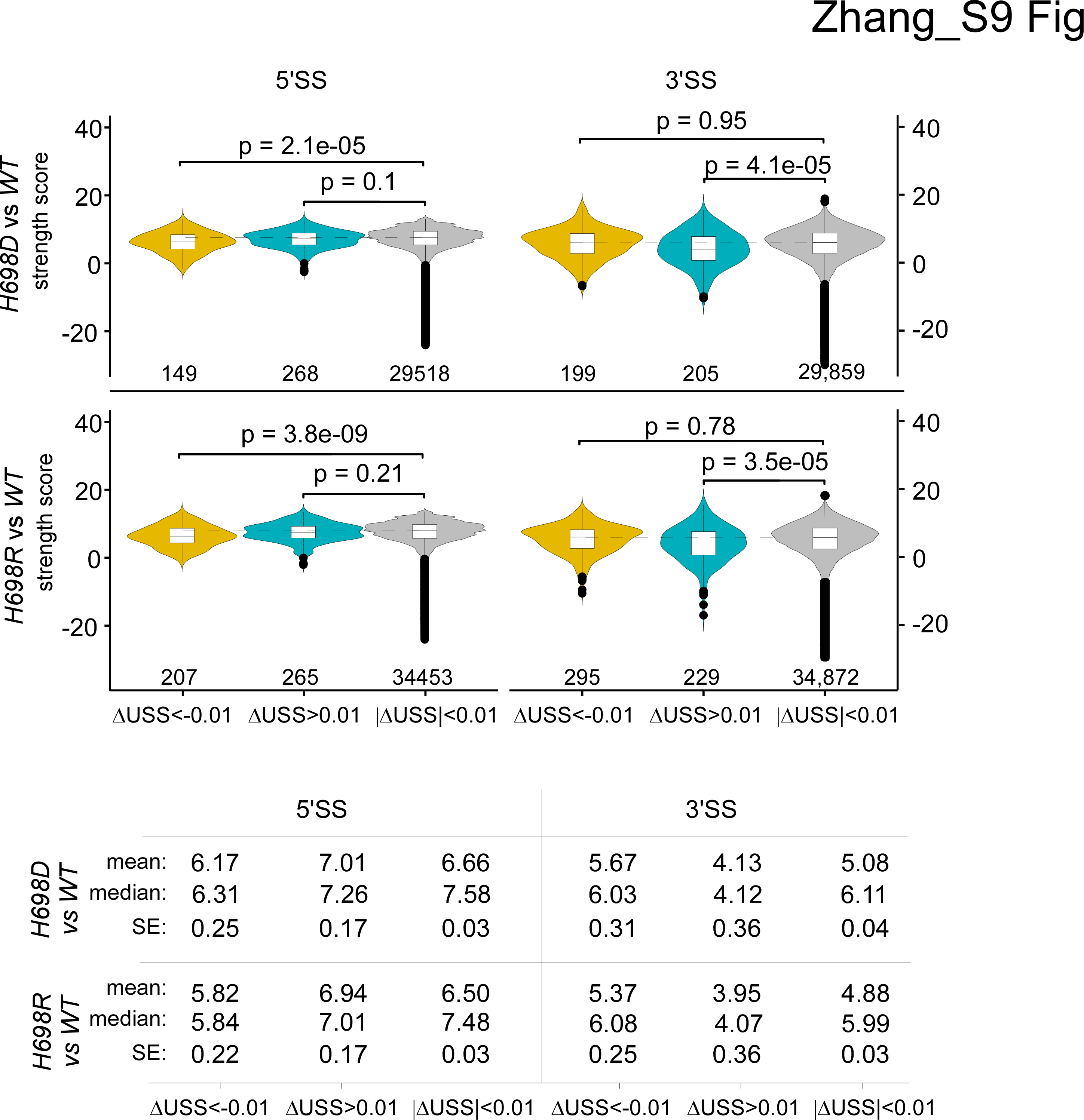

Supplement: S9 Fig — The 5′ and 3′SSs with |ΔUSS| > 0.01, FDR < 0.05 were screened in H698D and H698R and compared with the usage-not-changed SSs (|ΔUSS| < 0.01). Blue: ΔUSS > 0.01, brown: ΔUSS < -0.01, grey: |ΔUSS| < 0.01. Values of mean, median, and SE from each group are presented. (TIF) [file pgen.1009861.s009.tif]

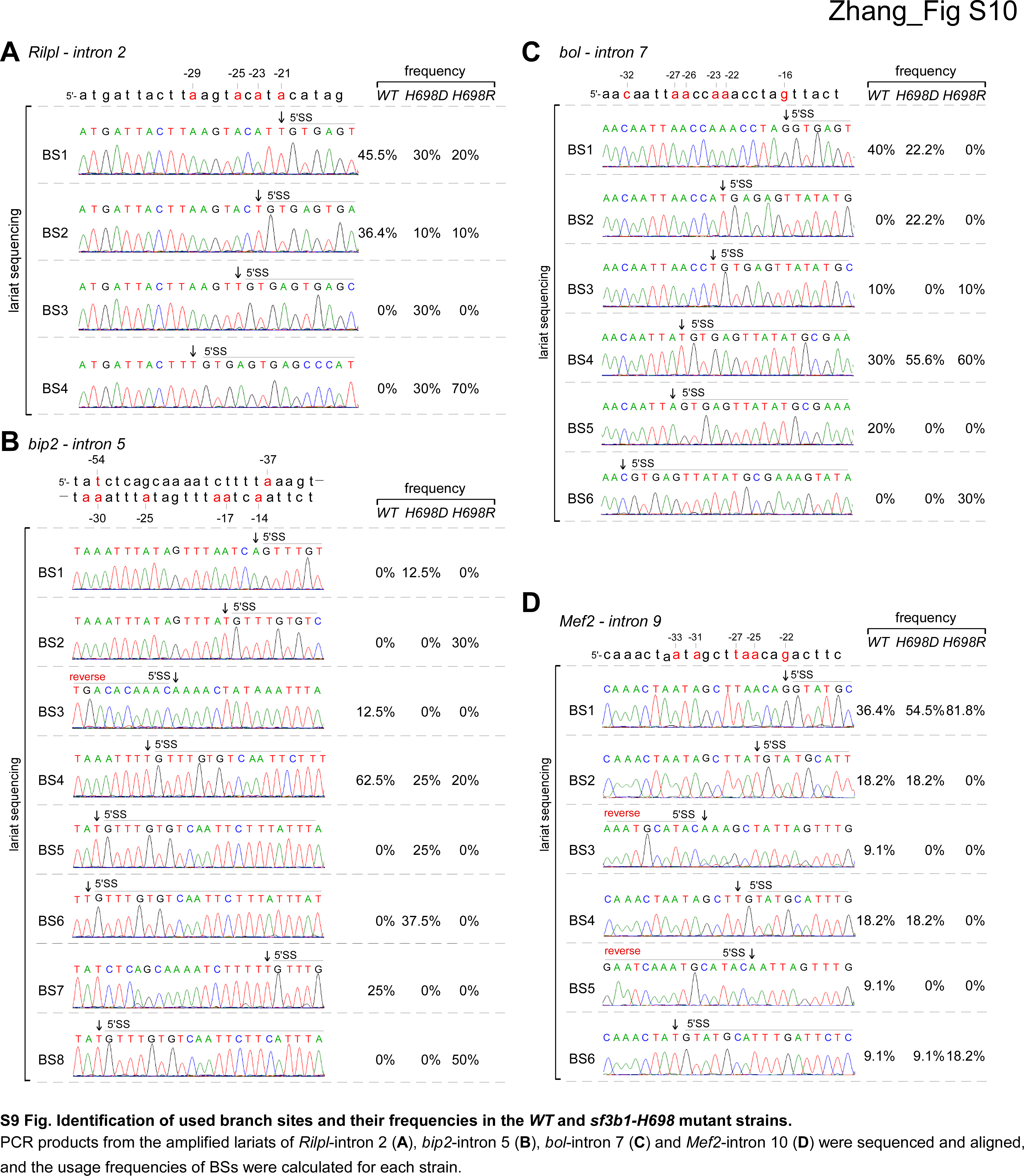

Supplement: S10 Fig — PCR products from the amplified lariats of Rilpl-intron 2 (A), bip2-intron 5 (B), bol-intron 7 (C) and Mef2-intron 10 (D) were sequenced and aligned, and the usage frequencies of BSs were calculated for each strain. (TIF) [file pgen.1009861.s010.tif]

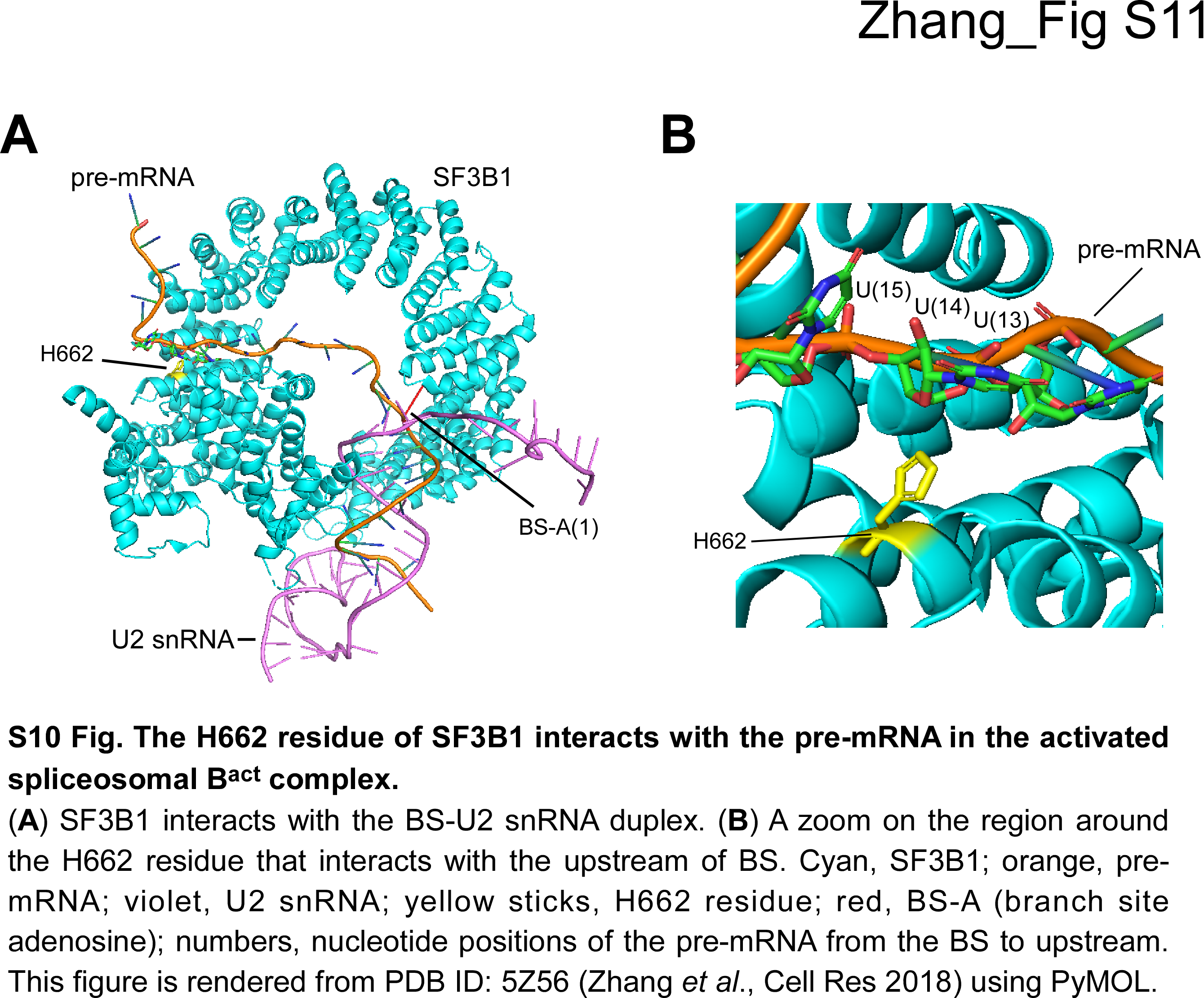

Supplement: S11 Fig — (A) SF3B1 interacts with the BS—U2 snRNA duplex. (B) A zoom on the region around the H662 residue that interacts with the upstream of BS. Cyan, SF3B1; orange, pre-mRNA; violet, U2 snRNA; yellow sticks, H662 residue; red, BS-A (branch site adenosine); numbers, nucleotide positions of the pre-mRNA from the BS to upstream. This figure is rendered from PDB ID: 5Z56 (Zhang et al., Cell Res 2018) using PyMOL. (TIF) [file pgen.1009861.s011.tif]
