# Supplementary material for: Two oppositely-charged sf3b1 mutations cause defective development, impaired immune response, and aberrant selection of intronic branch sites in Drosophila
Source: PLoS Genet. 2021 Nov 1;17(11):e1009861. doi: 10.1371/journal.pgen.1009861 (PMC8559932; doi:10.1371/journal.pgen.1009861)
Supplement: S1 Table — (DOCX) [file pgen.1009861.s012.docx]

**S1 Table. Summary of SF3b1 mutations in human cancers.**

| **Mutation sites** | **Tumor type** | **Mutation in patients** | **Reference** |
| --- | --- | --- | --- |
| K700E(44), K666N(6)/T(3)/E(2)/R(2), H662Q(8)/D(2), R625L(2)/C, E622D(4) | MDS | 79 / 582 | [[1](#_ENREF_1)] |
| K700E(97), K666R(9)/N(3)/Q(3)/T(2)/M, H662Q(15)/D/Y, E622D(12), R625L(6)/C(3)/G, D781G(3), E592K(2), A744P(2), E491G, R590K, N626D, V701F/I, G740R, A1188V | MDS | 150 / 533 | [[2](#_ENREF_2)] |
| K700E(59), E622(5), R625 (7), H662Q (7), K666 (13), I704 (3) | MDS, CLL, Breast, etc | 100 / 2,087 | [[3](#_ENREF_3)] |
| K700E(9), G742D(6), K666E(2), T663I (2), N626Y(2), Y623C, R625H, H662D, V701F, K741N, D894G | CLL | 27 / 279 | [[4](#_ENREF_4)] |
| K700E(7), G742D(2), N626H, Q903R, G740E, R625L, K741N | CLL | 14/91 | [[5](#_ENREF_5)] |
| D781G(2), K700E(30), K666N(6)/Q, H662Y(2)/Q, R625L/C/H/G, E622D | MDS | 47 / 317 | [[6](#_ENREF_6)] |
| D781G(2), K700E(21), R625C/L, E622D, K666N(2)/R(2)/M/T/Q, H662Q(2)/Y(2) | MDS | 37 / 221 | [[7](#_ENREF_7)] |
| K700E(36), R625C/L(7), K666R/T(6), H662Q(3), G740E/V(2), E622D | MDS-RS | 55 / 104 | [[8](#_ENREF_8)] |
| K700E(14), K666T/E/M, H662Q, E622D, R625L/C | MDS | 22 / 56 | [[9](#_ENREF_9)] |
| K700E(10), H662Q(2), K666N | CMML | 13 / 226 | [[10](#_ENREF_10)] |
| R625H(11)/C(7), E622D, Y623H | UVM | 11 / 23 | [[11](#_ENREF_11)] |
| R625H(9)/C(4)/P/L, K666T(2), K700E | UVM | 18 / 119 | [[12](#_ENREF_12)] |
| R625H(12)/C(5)/L/G | UVM | 19 / 102 | [[13](#_ENREF_13)] |
| G742D, K700E(10), K666Q(2), H662Q(2), R625C | MDS | 16 / 36 | [[14](#_ENREF_14)] |
| K700E(11), H662Q(4), K666Q/R(2), E622D(2), D781G, R625C | MDS | 24 / 154 | [[15](#_ENREF_15)] |
| K700E(33), K666N(5)/Q/T/E, R625C(2), A672D, E622D, H662Q/D, I704N | MDS | 48 / 479 | [[16](#_ENREF_16)] |
| K700N/E(50), H662D(2)/Q, G742D(14), K666S/N(2)/T/E(8), R625G/H/C(2), E622D(6) | CLL | 104 / 1,160 | [[17](#_ENREF_17)] |
| K700E(17), K666Q/E | Breast | 23 / 1,293 | [[18](#_ENREF_18)] |
| K700E(7), K666N, H662Q | MDS w/o RS | 9 / 129 | [[19](#_ENREF_19)] |
| G742D(4)/E, G740E, V701F, K700E(16), K666T(2), T663, H662R, R625C(8)/H(9), Y623C, E622D(3) | MDS, CLL, UVM, breast, SKCM | 47 / 99 | [[20](#_ENREF_20)] |
| K700E(12), K666N(3)/T(2)/E, H662Y, R625H(2), E622D(2), E592K, G605S | MDS | 25 / 304 | [[21](#_ENREF_21)] |
| R625H(8)/C(4)/G/L, K666M/T | UVM | 16 / 74 | [[22](#_ENREF_22)] |
| R625(5), N626 | UVM | 6 / 33 | [[23](#_ENREF_23)] |
| K700E(19), K666R(4)/T, T663I, H662Q(2)/D, N626D2, R625L3, Y623C, E622D5 | MDS-RS | 39 / 40 | [[24](#_ENREF_24)] |
| R625H(5)/C/S, E1105G | mucosal melanoma | 8 / 19 | [[25](#_ENREF_25)] |
| R625H (4) | Ocular melanoma | 4 / 11 | [[26](#_ENREF_26)] |
| K700E(3), D781G(2), H662D | MDS | 6 / 9 | [[27](#_ENREF_27)] |
| K7005(6), H662Q, R625C, E622D | MDS | 9 / 55 | [[28](#_ENREF_28)] |
| E622D, H662Q, K666T, K700E(6) | MDS-RS | 9 / 67 | [[29](#_ENREF_29)] |
| G742D(2), K741E/N, G740E, K700E(13), K666E, N626I(2), E622D | CLL | 22 /399 | [[30](#_ENREF_30)] |
| K700E(147), R625C/G/H/L(19), K666M/N/Q/R/T(12), H662D(7)/N, G740E/N | MDS | 193 / 255 | [[31](#_ENREF_31)] |
| K700E (6) | CLL | 6 / 12 | [[32](#_ENREF_32)] |
| K700E (74), K666 (5), N626 (3), R625 (2) | Breast | 90 / 5,366 | [[33](#_ENREF_33)] |
| R625H (45) | prolactinomas | 45 / 227 | [[34](#_ENREF_34)] |

**Notes:** Frequency of SF3b1 mutations in patients are extracted from below 34 reports.

MDS: myelodysplastic syndromes; CLL: chronic lymphocytic leukemia; MDS-RS: myelodysplastic syndromes with ring sideroblasts; CMML: chronic myelomonocytic leukemia; SKCM: skin melanoma; UVM: Uveal Melanoma

**Reference**:

1. Yoshida K, Sanada M, Shiraishi Y, Nowak D, Nagata Y, et al. (2011) Frequent pathway mutations of splicing machinery in myelodysplasia. Nature 478: 64-69.

2. Malcovati L, Papaemmanuil E, Bowen DT, Boultwood J, Della Porta MG, et al. (2011) Clinical significance of SF3B1 mutations in myelodysplastic syndromes and myelodysplastic/myeloproliferative neoplasms. Blood 118: 6239-6246.

3. Papaemmanuil E, Cazzola M, Boultwood J, Malcovati L, Vyas P, et al. (2011) Somatic SF3B1 mutation in myelodysplasia with ring sideroblasts. New England Journal of Medicine 365: 1384–1395.

4. Quesada V, Conde L, Villamor N, Ordonez GR, Jares P, et al. (2011) Exome sequencing identifies recurrent mutations of the splicing factor SF3B1 gene in chronic lymphocytic leukemia. Nat Genet 44: 47-52.

5. Wang L, Lawrence MS, Wan Y, Stojanov P, Sougnez C, et al. (2011) SF3B1 and other novel cancer genes in chronic lymphocytic leukemia. N Engl J Med 365: 2497-2506.

6. Damm F, Thol F, Kosmider O, Kade S, Loffeld P, et al. (2012) SF3B1 mutations in myelodysplastic syndromes: clinical associations and prognostic implications. Leukemia 26: 1137-1140.

7. Damm F, Kosmider O, Gelsi-Boyer V, Renneville A, Carbuccia N, et al. (2012) Mutations affecting mRNA splicing define distinct clinical phenotypes and correlate with patient outcome in myelodysplastic syndromes. Blood 119: 3211-3218.

8. Cui R, Gale RP, Xu Z, Qin T, Fang L, et al. (2012) Clinical importance of SF3B1 mutations in Chinese with myelodysplastic syndromes with ring sideroblasts. Leuk Res 36: 1428-1433.

9. Visconte V, Makishima H, Jankowska A, Szpurka H, Traina F, et al. (2012) SF3B1, a splicing factor is frequently mutated in refractory anemia with ring sideroblasts. Leukemia 26: 542-545.

10. Patnaik MM, Lasho TL, Finke CM, Hanson CA, Hodnefield JM, et al. (2013) Spliceosome mutations involving SRSF2, SF3B1, and U2AF35 in chronic myelomonocytic leukemia: prevalence, clinical correlates, and prognostic relevance. Am J Hematol 88: 201-206.

11. Martin M, Masshofer L, Temming P, Rahmann S, Metz C, et al. (2013) Exome sequencing identifies recurrent somatic mutations in EIF1AX and SF3B1 in uveal melanoma with disomy 3. Nat Genet 45: 933-936.

12. Furney SJ, Pedersen M, Gentien D, Dumont AG, Rapinat A, et al. (2013) SF3B1 mutations are associated with alternative splicing in uveal melanoma. Cancer Discov 3: 1122-1129.

13. Harbour JW, Roberson ED, Anbunathan H, Onken MD, Worley LA, et al. (2013) Recurrent mutations at codon 625 of the splicing factor SF3B1 in uveal melanoma. Nat Genet 45: 133-135.

14. Seo JYL, Ki-O; Kim, Sun-Hee; Kim, Kihyun; Jung, Chul Won; Jang, Jun Ho; Kim, Hee-Jin (2014) Clinical significance of SF3B1 mutations in Korean patients with myelodysplastic syndromes and myelodysplasia/myeloproliferative neoplasms with ring sideroblasts. Annals of Hematology 93: 603–608.

15. Mian SA, Smith AE, Kulasekararaj AG, Kizilors A, Mohamedali AM, et al. (2013) Spliceosome mutations exhibit specific associations with epigenetic modifiers and proto-oncogenes mutated in myelodysplastic syndrome. Haematologica 98: 1058-1066.

16. Lin CC, Hou HA, Chou WC, Kuo YY, Wu SJ, et al. (2014) SF3B1 mutations in patients with myelodysplastic syndromes: the mutation is stable during disease evolution. Am J Hematol 89: E109-115.

17. Jeromin S, Weissmann S, Haferlach C, Dicker F, Bayer K, et al. (2014) SF3B1 mutations correlated to cytogenetics and mutations in NOTCH1, FBXW7, MYD88, XPO1 and TP53 in 1160 untreated CLL patients. Leukemia 28: 108-117.

18. Maguire SL, Leonidou A, Wai P, Marchio C, Ng CK, et al. (2015) SF3B1 mutations constitute a novel therapeutic target in breast cancer. J Pathol 235: 571-580.

19. Kang MG, Kim HR, Seo BY, Lee JH, Choi SY, et al. (2015) The prognostic impact of mutations in spliceosomal genes for myelodysplastic syndrome patients without ring sideroblasts. BMC Cancer 15: 484.

20. Darman RB, Seiler M, Agrawal AA, Lim KH, Peng S, et al. (2015) Cancer-Associated SF3B1 Hotspot Mutations Induce Cryptic 3' Splice Site Selection through Use of a Different Branch Point. Cell Rep 13: 1033-1045.

21. Wu L, Song L, Xu L, Chang C, Xu F, et al. (2016) Genetic landscape of recurrent ASXL1, U2AF1, SF3B1, SRSF2, and EZH2 mutations in 304 Chinese patients with myelodysplastic syndromes. Tumour Biol 37: 4633-4640.

22. Alsafadi S, Houy A, Battistella A, Popova T, Wassef M, et al. (2016) Cancer-associated SF3B1 mutations affect alternative splicing by promoting alternative branchpoint usage. Nat Commun 7: 10615.

23. Royer-Bertrand B, Torsello M, Rimoldi D, El Zaoui I, Cisarova K, et al. (2016) Comprehensive Genetic Landscape of Uveal Melanoma by Whole-Genome Sequencing. Am J Hum Genet 99: 1190-1198.

24. Mortera-Blanco T, Dimitriou M, Woll PS, Karimi M, Elvarsdottir E, et al. (2017) SF3B1-initiating mutations in MDS-RSs target lymphomyeloid hematopoietic stem cells. Blood 130: 881-890.

25. Hintzsche JD, Gorden, N. T., Amato, C. M., Kim, J., Wuensch, K. E., Robinson, S. E., … Robinson, W. A. (2017) Whole-exome sequencing identifies recurrent SF3B1 R625 mutation and comutation of NF1 and KIT in mucosal melanoma. Melanoma Research 27(3): 189–199.

26. Rose AM, Luo R, Radia UK, Kalirai H, Thornton S, et al. (2018) Detection of mutations in SF3B1, EIF1AX and GNAQ in primary orbital melanoma by candidate gene analysis. BMC Cancer 18: 1262.

27. Zhang J, Ali AM, Lieu YK, Liu Z, Gao J, et al. (2019) Disease-Causing Mutations in SF3B1 Alter Splicing by Disrupting Interaction with SUGP1. Mol Cell 76: 82-95 e87.

28. Yimpak P, Tantiworawit A, Rattanathammethee T, Angsuchawan S, Laowatthanapong S, et al. (2019) Alteration of SF3B1 and SRSF2 Genes in Myelodysplastic Syndromes Patients in Upper Northern Thailand. Asian Pac J Cancer Prev 20: 1215-1221.

29. Mizuta S, Yamane N, Komai T, Koba Y, Ukyo N, et al. (2019) Evaluation of SF3B1 Mutation Screening by High-Resolution Melting Analysis and its Clinical Utility for Myelodysplastic Syndrome with Ring Sideroblasts at the Point of Diagnosis. Lab Med 50: 254-262.

30. Miao Y, Zou YX, Gu DL, Zhu HC, Zhu HY, et al. (2019) SF3B1 mutation predicts unfavorable treatment-free survival in Chinese chronic lymphocytic leukemia patients. Ann Transl Med 7: 176.

31. Cai YN, Xu ZF, Li B, Qin TJ, Pan LJ, et al. (2020) [Features and clinical significance of gene mutations in patients with myelodysplastic syndromes with ring sideroblasts]. Zhonghua Xue Ye Xue Za Zhi 41: 379-386.

32. Liu Z, Yoshimi A, Wang J, Cho H, Chun-Wei Lee S, et al. (2020) Mutations in the RNA Splicing Factor SF3B1 Promote Tumorigenesis through MYC Stabilization. Cancer Discov 10: 806-821.

33. Liu B, Liu Z, Chen S, Ki M, Erickson C, et al. (2020) Mutant SF3B1 promotes AKT and NF-kB driven mammary tumorigenesis. J Clin Invest.

34. Li C, Xie W, Rosenblum JS, Zhou J, Guo J, et al. (2020) Somatic SF3B1 hotspot mutation in prolactinomas. Nat Commun 11: 2506.
