# Supplementary material for: Two oppositely-charged sf3b1 mutations cause defective development, impaired immune response, and aberrant selection of intronic branch sites in Drosophila
Source: PLoS Genet. 2021 Nov 1;17(11):e1009861. doi: 10.1371/journal.pgen.1009861 (PMC8559932; doi:10.1371/journal.pgen.1009861)
Supplement: S2 Table — (DOCX) [file pgen.1009861.s013.docx]

**Zhang _Table S2**

**S2 Table. RNA-seq samples and reads in this study.**

| **Samples** | **Clean reads** | **Mapped reads** | **Mapping ratio (%)** |
| --- | --- | --- | --- |
| WT_1 | 44,167,946 | 42,158,459 | 95.45 |
| WT_2 | 51,216,548 | 48,913,617 | 95.50 |
| WT_3 | 52,503,542 | 50,153,380 | 95.52 |
| H698D_1_1 | 53,562,494 | 51,105,174 | 95.41 |
| H698D_1_2 | 49,862,982 | 47,533,789 | 95.33 |
| H698D_1_3 | 50,566,752 | 48,130,552 | 95.18 |
| H698D_2_1 | 49,478,120 | 48,328,836 | 97.68 |
| H698D_2_2 | 35,790,704 | 35,047,704 | 97.92 |
| H698D_2_3 | 52,649,366 | 51,463,329 | 97.75 |
| H698R_1_1 | 51,200,284 | 48,791,420 | 95.30 |
| H698R_1_2 | 48,817,882 | 46,726,621 | 95.72 |
| H698R_1_3 | 44,167,946 | 42,158,459 | 95.45 |
| H698R_2_1 | 42,020,276 | 41,062,919 | 97.72 |
| H698R_2_2 | 56,013,216 | 54,819,272 | 97.87 |
| H698R_2_3 | 41,114,388 | 40,131,265 | 97.61 |

Notes: mRNA from two lines of each *sf3b1* mutant strain and the *WT* strain (5905) were sequenced in triplicates.
