# Supplementary material for: Two oppositely-charged sf3b1 mutations cause defective development, impaired immune response, and aberrant selection of intronic branch sites in Drosophila
Source: PLoS Genet. 2021 Nov 1;17(11):e1009861. doi: 10.1371/journal.pgen.1009861 (PMC8559932; doi:10.1371/journal.pgen.1009861)
Supplement: S7 Table — (DOCX) [file pgen.1009861.s018.docx]

**Zhang_Table S7**

**S7 Table. Primers used in this study**

| **Primers** | **Sequences** | **Notes** |
| --- | --- | --- |
| ZB001 | 5'_TTCG TCCTGGCAAGCGCGGCACAC | sgRNA Sf3b1 ex3 F |
| ZB002 | 5'_AAAC GTGTGCCGCGCTTGCCAGGA | sgRNA Sf3b1 ex3 R |
| ZB003 | 5'_AGACTCCAAAGCCAGATCGC | donor L Sf3b1 ex3 F |
| ZB004 | 5'_TTGATGCCAGTATCCCGCGCTTGC | donor L Sf3b1 ex3 R (H698D) |
| ZB005 | 5'_GCAAGCGCGGGATACTGGCATCAA | donor R Sf3b1 ex3 F (H698D) |
| ZB006 | 5'_CAGGGAGCACGGTAAAAGGT | donor R Sf3b1 ex3 R |
| ZB007 | 5'_TTGATGCCAGTACGCCGCGCTTGC | donor L Sf3b1 ex3 R (H698R) |
| ZB008 | 5'_GCAAGCGCGGCGTACTGGCATCAA | donor R Sf3b1 ex3 F (H698R) |
| ZB009 | 5'_CATTGGAGGACCAAGAGCGT | screening primer F |
| ZB010 | 5'_CAATTTTGATGCCAGT GTG | screening primer R (H698) |
| ZB011 | 5'_GACAATTTTGATGCCAGT ATC | screening primer R (H698D) |
| ZB012 | 5'_CAATTTTGATGCCAGT ACG | screening primer R (H698R) |
| ZB013 | 5'_TGACCATCCGCCCAGCATACAG | rp49 |
| ZB014 | 5'_GTTCTCTTGAGAACGCAGGCGA | rp49 |
| ZB015 | 5'_GCCAAGGGAACCATTGTTGT | Cyp6a17 ex2 F |
| ZB016 | 5'_TCCAAATGGAAGCCAAGTGC | Cyp6a17 ex2 R |
| ZB017 | 5'_TCGGCTCAAATGTGCTGATG | Cyp12d1-p ex4 F |
| ZB018 | 5'_GTCCAAAGCCAAAGGGAAGG | Cyp12d1-p ex4 R |
| ZB019 | 5'_TTCCAGGAGCATGCCAACTA | Cyp4e3 ex3 F |
| ZB020 | 5'_AATGCCTGGACAATGGAGGA | Cyp4e3 ex3 R |
| ZB021 | 5'_TACCACATGGACTCCCTGGGCT | PGRP-S ex1 F |
| ZB022 | 5'_GCGGATCTCGTTCCAGATGT | PGRP-S ex1 R |
| ZB023 | 5'_ACATCCAACCAGCAGCACAT | mxt ex3 F |
| ZB024 | 5'_TCTGCACCAAACGCTCCTTG | mxt ex5 R |
| ZB025 | 5'_ACTATACGCCTGCTGTTGGG | tea ex10 F |
| ZB026 | 5'_CTTCAGCAGGTTGGGTCGAT | tea ex11 R |
| ZB027 | 5'_ACAATACCATCCTGA CCGCA | Rtnl1 ex1 F |
| ZB028 | 5'_GCACACTTCTTCGTTAGCACT | Rtnl1 ex2 R |
| ZB029 | 5'_ATGTGCTACCAGCTGGCAGT | LUBEL ex1 F |
| ZB030 | 5'_TCGTC TGGGTTTTGCCGTCACT | LUBEL ex2 R |
| ZB031 | 5'_AACCAGCCGAAACTCACAGT | Gprk2 ex1 F |
| ZB032 | 5'_TATTTGGCAGCAGGGTCT CG | Gprk2 ex3 R |
| ZB033 | 5'_ACCACTCATTCGACACATTT | CG3694(Gγ30A) ex1 F |
| ZB034 | 5'_TGGCCCGTCTATCGATGCTA | CG3694(Gγ30A) ex2 R |
| ZB035 | 5'_ATCGCAATTGGAAACTGGACTT | Vha-100 ex1 F |
| ZB036 | 5'_GGTTTCGCCCAGCTCAGATAC | Vha-100 ex2 R |
| ZB037 | 5'_CACATCACACCCACTCCA G | Mef2 ex9 F |
| ZB038 | 5'_TATTCTCGGCCGTTTGTGGT | Mef2 ex11 R |
| ZB039 | 5'_CACCTCCACATCCACCTCAG | CG31550 ex2 F |
| ZB040 | 5'_TTAGCTTCTGATGCTCCCG C | CG31550 ex4 R |
| ZB041 | 5'_TGCGTCACTCATATCGGCTC | CG2316 ex4 F |
| ZB042 | 5'_CGATACCAGCTGTCCGAACT | CG2316 ex6 F |
| ZB043 | 5'_GCAACAGCAGCAACAACTTCA T CA | aPKC ex9 F |
| ZB044 | 5'_TGTGT C TGTTG TTGA GAA A G GA | aPKC ex10 R |
| ZB045 | 5'_GGGTATTGCCAACAATTTAAG | ND-15 ex1 F |
| ZB046 | 5'_ATGGAAGCGCATCAGTTGCTT | ND-15 ex2 R |
| ZB047 | 5'_GCTCAGT C T TGTAC GAAC CGT | Bacc ex1 F |
| ZB048 | 5'_GT TCCGTAGCAGCCGACAT A | Bacc ex2 R |
| ZB049 | 5'_CATCCTGAGGCCA GAAGGTC | CG17977 ex2 F |
| ZB050 | 5'_CTCCTCCGTAAG TCGTGG TT | CG17977 ex3 R |
| ZB051 | 5'_CCAGCACGGTCACATTCG A T | CG4658 ex1 F |
| ZB052 | 5'_TGGA A CTCGT GTTG CCC A T C | CG4658 ex2 R |
| ZB053 | 5'_CGTACCCCCACCACACAAAT | CG31446 ex2 F |
| ZB054 | 5'_ATCCGGATGGTGCATAGCAA | CG31446 ex3 R |
| ZB055 | 5'_CAGCGTCACTTCTCCAGCTC | Rilpl ex2 F |
| ZB056 | 5'_CTCTTCTTCCAGGGCGCATC | Rilpl ex3 R |
| ZB057 | 5'_AAAGGATGTAACAGAAATGGGCTCACTC | Rilpl lariat P2' R |
| ZB058 | 5'_GTTCATAAATTCTTGTTTTCTCTGGTGA | Rilpl lariat P1' R |
| ZB059 | 5'_aacgaacaagcattttcagcCTGA | Rilpl lariat P1 F |
| ZB060 | 5'_ctggtaaatggcacaattggg | Rilpl lariat P2 F |
| ZB061 | 5'_ACACCAACTGGATTAACGTCG | bip2 ex5 F |
| ZB062 | 5'_GCGGCACCTAACGATGGATT | bip2 ex6 R |
| ZB063 | 5'_TATCT GATCC AAATA CCACA ACA | bip2 lariat P2' R |
| ZB064 | 5'_GCTTA TCAGC TCCGG CGTATC | bip2 lariat P1' R |
| ZB065 | 5'_GACTAATTATAACATCGAAGCC | bip2 lariat P1 F |
| ZB066 | 5'_ACATGTAAAGCAGCCAAATC | bip2 lariat P2 F |
| ZB067 | 5'_ACCCATGGATCAGTTCGCAG | bol ex7 F |
| ZB068 | 5'_GAAGGTGGGTAGATGGCTGG | bol ex8 R |
| ZB069 | 5'_TCAGA GAAAC GATGA AGACCC | bol lariat P2' R |
| ZB070 | 5'_AACAAA TGTCAAT AAAACC AAGTG | bol lariat P1' R |
| ZB071 | 5'_tttcattacgctctctttcttaactcg | bol lariat P1 F |
| ZB072 | 5'_acaactacaaccaaaacaccacca | bol lariat P2 F |
| ZB073 | 5'_CAAGCACGAGGGAGAAGTCA | Mef2 lariat P2' R |
| ZB074 | 5'_AAACGGCAGCAATTTTCGCAAGCG | Mef2 lariat P1' R |
| ZB075 | 5'_ggtttgcctgcctttcgttt | Mef2 lariat P1 F |
| ZB076 | 5'_tccgcctgcctttgtcatac | Mef2 lariat P2 F |
